# Pan-cancer drivers of metastasis

**DOI:** 10.1186/s12943-024-02182-w

**Published:** 2025-01-02

**Authors:** Ryan Lusby, Engin Demirdizen, Mohammed Inayatullah, Paramita Kundu, Oscar Maiques, Ziyi Zhang, Mikkel Green Terp, Victoria Sanz-Moreno, Vijay K. Tiwari

**Affiliations:** 1 Wellcome-Wolfson Institute for Experimental Medicine, School of Medicine, Dentistry & Biomedical Science, Queens University Belfast, Belfast, BT9 7BL UK; 2 Institute for Molecular Medicine, University of Southern Denmark, Campusvej 55, 5230 Odense M, Denmark; 3 The Breast Cancer Now Toby Robins Research Centre, The Institute of Cancer Research, London, SW3 6JB UK; 4 Barts Cancer Institute, Queen Mary University of London, John Vane Science Building, Charterhouse Square, London, EC1M 6BQ UK; 5 Patrick G Johnston Centre for Cancer Research, School of Medicine, Dentistry & Biomedical Science, Queen’s University Belfast, BT9 7AE Belfast, UK; 6 Danish Institute for Advanced Study (DIAS), University of Southern Denmark, Campusvej 55, 5230 Odense M, Denmark; 7 Department of Clinical Genetics, Odense University Hospital, 5000 Odense C, Denmark

**Keywords:** Transcription Factors, Gene regulation, Single-cell heterogeneity, Metastasis, Cancer

## Abstract

Metastasis remains a leading cause of cancer-related mortality, irrespective of the primary tumour origin. However, the core gene regulatory program governing distinct stages of metastasis across cancers remains poorly understood. We investigate this through single-cell transcriptome analysis encompassing over two hundred patients with metastatic and non-metastatic tumours across six cancer types. Our analysis revealed a prognostic core gene signature that provides insights into the intricate cellular dynamics and gene regulatory networks driving metastasis progression at the pan-cancer and single-cell level. Notably, the dissection of transcription factor networks active across different stages of metastasis, combined with functional perturbation, identified SP1 and KLF5 as key regulators, acting as drivers and suppressors of metastasis, respectively, at critical steps of this transition across multiple cancer types. Through in vivo and in vitro loss of function of SP1 in cancer cells, we revealed its role in driving cancer cell survival, invasive growth, and metastatic colonisation. Furthermore, tumour cells and the microenvironment increasingly engage in communication through WNT signalling as metastasis progresses, driven by SP1. Further validating these observations, a drug repurposing analysis identified distinct FDA-approved drugs with anti-metastasis properties, including inhibitors of WNT signalling across various cancers.

**Supplementary Information:**

The online version contains supplementary material available at 10.1186/s12943-024-02182-w.

## Introduction

Cancer metastasis dramatically reduces survival and is the greatest cause of death for these patients [1, 2]. Metastasis involves cancer cells leaving the primary tumour and colonising distant organs [3, 4]. During the early stages of metastasis, cancer cells acquire the epithelial–mesenchymal transition (EMT) program to become mobile and invasive [5, 6]. Furthermore, the metastatic progression is influenced by complex interactions of cancer cells with the microenvironment at the primary and secondary sites, manifesting different cell fates [3, 4, 7]. Despite over 200 drugs approved in the last six decades targeting various aspects of this process, the overall survival in metastatic disease remains poor [8]. Moreover, while all cancer types share hallmarks of metastasis, whether a treatment could target this process irrespective of the tissue origin is unclear [9, 10]. Although combination therapy or neoadjuvant therapy can have a therapeutic benefit in several cancers, their drawbacks include triggering of the metastatic cascade and drug resistance [11–13]. Elucidating the potential of when a patient is likely to metastasize and targeting it timely and effectively is the biggest unmet need in clinical practice.

Previous studies have focused on investigating features of metastatic potential in specific cancers with several signatures showing pre-clinical utility in their corresponding cancer types [14, 15]. For example, in breast cancer, many studies have identified distinct gene expression signatures that can predict metastatic progression [16–18]. Similar advances have been made for lung, colorectal and prostate cancers [19–21]. However, these signatures only apply to the cancer type which they were identified in and thus show no versatile utility for other cancer types [22]. This has further prevented identifying common therapeutic targets for preventing metastasis.

In recent years, there has been a shift in the examination of the molecular mechanisms of metastasis at the pan-cancer level, which has uncovered subtypes of metastasis that transcend primary tumours and helped inform precision medicine [23–25]. For example, a recent paper investigating the genomic landscape of primary and metastatic tumours at the pan-cancer level found metastatic lesions to be less heterogeneous than reported for primary tumours, implying that shared transcriptional programs across metastatic tumours might exist [26]. However, these studies applied bulk sequencing techniques, which mask the heterogeneity within the tumour and tumour microenvironment [27]. This is a key limitation, as metastasis is a multi-step process, involving continuous communication within subpopulations of the tumour and with the microenvironment. This information is masked when averaging information in bulk sequencing approaches [27]. These approaches have consequently not been able to help current clinical practices detect early disseminating cells, resulting in underestimation of a patient’s current metastatic state and risk.

To address this unmet need and overcome the limitation, we conducted a pan-cancer single-cell transcriptome analysis, involving over 200 patients with metastatic and non-metastatic tumours across six cancer types. Our research has identified a core gene signature that effectively detects disseminating cancer cells and elucidates the cellular dynamics and gene regulatory networks driving stepwise metastasis progression at both the pan-cancer and single-cell levels. Notably, we dissected transcription factor networks active across various stages of metastasis. Through functional perturbation, we identified SP1 and KLF5 as crucial regulators, acting as drivers and suppressors of metastasis, respectively, across multiple cancer types. Additionally, we found that tumour cells and the microenvironment increasingly communicate via WNT signalling as metastasis begins, driven by SP1. Furthermore, a drug repurposing analysis followed by in vitro validation identified FDA-approved drugs with anti-metastasis properties, including inhibitors of WNT signalling, across various cancers.

## Results

### A core gene signature provides insights into metastatic potential across multiple cancer types

**Fig. 1:**
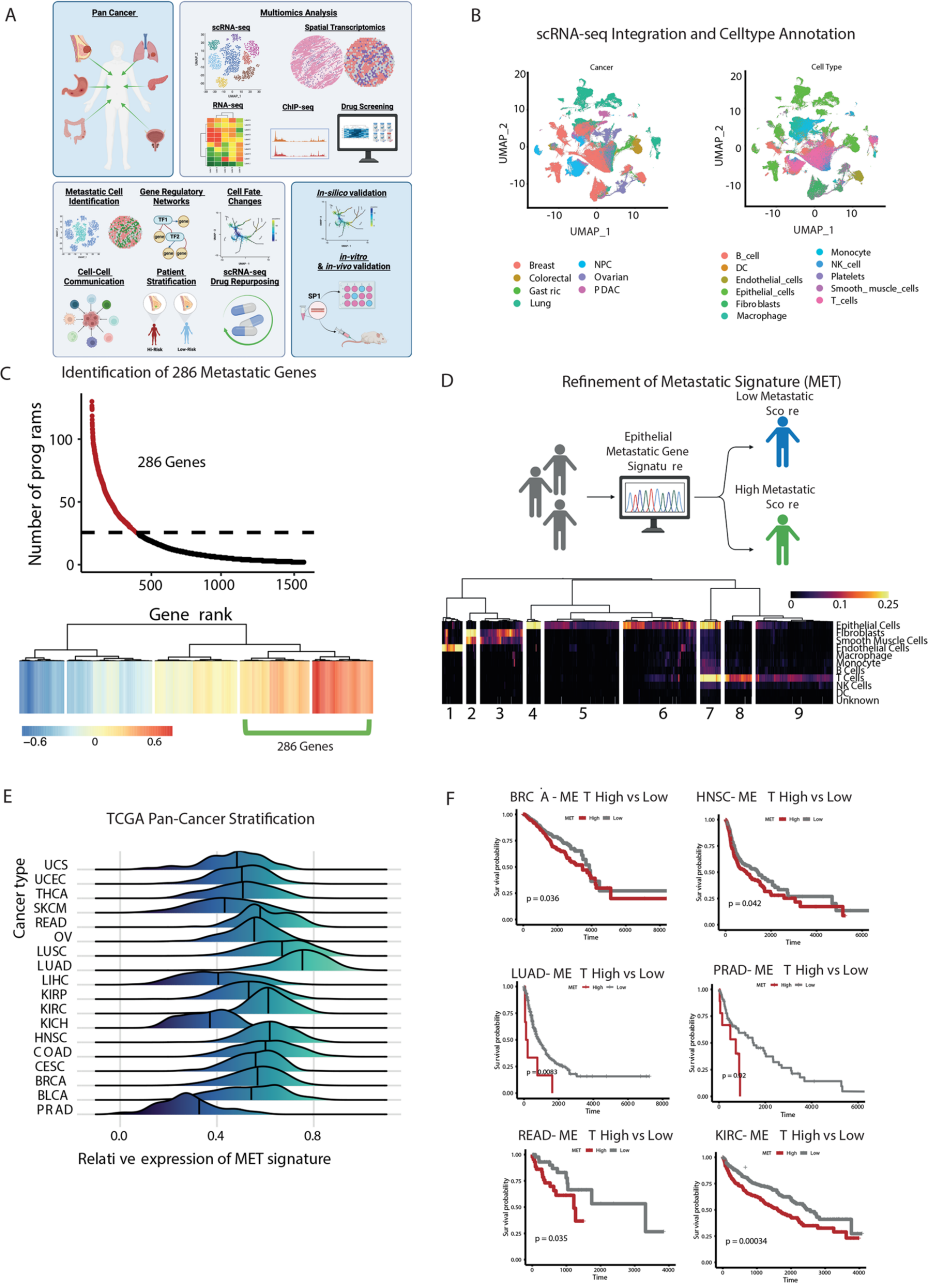
Defining the core transcriptional landscape driving pan-cancer metastasis. **A** Graphical overview of the study, highlighting the cancer types examined, the multi-omics data utilised, the in-silico analysis methods employed, and the validation approaches for in silico findings. **B** UMAP projection of pan-cancer single-cell RNA-seq (scRNA-seq) data, annotated by cancer types and cell types. **C** The top panel shows the number of programs associated with the expression of metastatic gene lists, while the bottom panel presents the clustering analysis of genes frequently associated with 25 or more programs across all samples, ranked by their association with the number of archetypes. **D** The top panel illustrates the aim to define a refined epithelial cell type–specific signature from 286 genes, and the bottom panel displays the cell type specificity scores of each metastatic gene across different cell types, with clusters annotated to highlight cluster-specific expression of the signature. **E** Metastatic scoring of each TCGA pan-cancer patient, stratifying them into high and low metastatic potential groups. **F** Kaplan–Meier survival plot of patients stratified by metastatic potential genes in the TCGA pan-cancer cohort

Given the known shared cellular hallmarks of metastasis across cancers, we hypothesised that a conserved metastatic signature may exist across multiple cancer types irrespective of tissue origin. To test this hypothesis, we curated single-cell transcriptome (scRNA-seq) data from 17 studies that encompassing 222 patients across six different cancer types (colorectal, gastric, lung, nasopharyngeal (NPC), ovarian, pancreatic ductal adenocarcinoma (PDAC), breast) (Supplementary Table 1) all of which are known to have a high risk of metastasis. After removing samples with fewer than 100 malignant cells, based on the results of applying inferCNV on each, we integrated each sample using Seurat, resulting in a pan-cancer dataset comprised of expression profiles of more than 1.2 million (1,237,224) cancer cells from 266 tumour samples (Fig. 1A-B).

Our primary objective was to identify specific groups of cells, regardless of their cell type, with a high potential to metastasize in patients across different types of cancer in our pan-cancer scRNA-seq. To do this, we started by examining the Human Cancer Metastasis Database in which they have a list of 2,183 genes available to download that have been linked to metastasis in various types of cancer [28]. From this database, we split the downloaded gene list into two lists, in which 1,426 genes were linked to metastasis based on one publication [28], and 753 genes were referred by multiple publications [28].

To create a core set of genes that can effectively identify metastatic cells, we performed a detailed analysis on each patient's sample utilizing multiresolution archetypal analysis from the ACTIONet R package [29]. This enabled us to identify common cells across different types of cancer and different patients based on gene expression patterns related to each metastatic gene list. Furthermore, this approach would enable us to select relevant genes, in the context of scRNA-seq, from a list of genes obtained from various omics-methods and cancer types as detailed in their publication [28].

To identify archetype programs with high metastatic potential, we applied our metastatic gene sets to score each archetype, using UCell, across the scRNA-seq data from patients. Specifically, we evaluated the expression levels of genes from each metastatic gene set within each archetype program. Archetypes exhibiting high expression of multiple genes from both sets were deemed to have increased metastatic potential. Supplementary Fig. 1A illustrates representative results for lung and breast cancer patients, where archetypes with high scores (indicated in red) were selected for further analysis. These high-scoring archetype programs, enriched with numerous metastasis-associated genes, likely represent cell subpopulations with a greater propensity for metastasis, as indicated by their shared transcriptomic signatures related to metastatic activity. This approach allowed us to narrow down specific archetype programs potentially linked to metastatic processes.

Next, we ranked the archetypes based on their UCell scores and concentrated on those with the highest UCell scores, as these are most likely associated with metastasis. We extracted genes present in 25 or more archetypes across all patients from these top-scoring archetypes. This threshold was determined by the inflection point of our curve analysis (Fig. 1C), ensuring that we selected genes consistently expressed in a substantial number of archetypes. We then performed linear regression analysis based on the number of archetypes in which each gene was expressed. We identified the top-ranking genes that define these archetype programs by clustering the genes according to this metric. This analysis culminated in a core metastatic signature of 286 genes (Fig. 1C), representing a key set of genes implicated in metastatic progression. Interestingly, these signature genes were associated with important processes related to metastasis, such as cell adhesion, regulation of cell proliferation, and epithelial cell differentiation (Supplementary Fig. 1B) [30].

Next, we scored each patient in our pan-cancer scRNA-seq data for a shared high expression of these 286 genes to identify those with high metastatic potential and to ensure that a specific cancer type was not driving the identified gene list. Interestingly, the outcome showed that this signature could rank each patient from high or low metastatic potential with no clear patterns in tumour type driving this scoring (Supplementary Fig. 1C).

We extracted available metadata on tumour stages for each patient to further investigate the relationship between our ranking and clinical parameters. Tumours were categorized as either above or below stage III, and we performed a correlation analysis between the tumour stage and the ranking scores derived from the expression of the 286 signature genes. The analysis revealed a weak positive correlation (r = 0.134) between the ranking scores and tumour stage (Supplementary Fig. 1D). This modest association aligns with our expectations, given that the ranking was based on the entire gene expression profile rather than a subset specifically optimized for predicting metastatic potential. While higher ranking scores tend to be associated with more advanced tumour stages, this ranking is not fully predictive.

Next, we aimed to validate the robustness of our gene signature using bulk RNA-seq data, accompanied by patient relapse-free survival information. However, bulk RNA-seq data can be influenced by a mixture of cell types, potentially skewing the results. We hypothesized that a gene signature specific to cancer epithelial cells, the main drivers of tumour behaviour, would provide more accurate prognostic information. To address this, we calculated a cell-type specificity score by evaluating the expression of each of the 286 metastasis-associated genes across all cell types. We then averaged these scores across tumours and visualized them in a heatmap, revealing nine distinct clusters based on gene expression patterns (Fig. 1D).

Since many genes were not specific to epithelial cells, we focused on clusters 4 and 5, which exhibited high expression in epithelial cells. This refinement yielded 177 genes with high epithelial specificity and minimal expression in other cell types (Fig. 1D). This subset provided a more targeted gene signature relevant to cancer epithelial cells. Gene ontology (GO) analysis of these 177 genes revealed their involvement in migratory processes and B cell activation (Supplementary Fig. 1E), both of which are critical in cancer progression and metastasis. We also conducted GO analysis on the remaining 109 genes from the original 286 that were not epithelial-specific. These genes were enriched in pathways related to extracellular matrix organization, angiogenesis, and blood vessel development, highlighting their significant roles in supporting the tumour microenvironment and metastatic processes (Supplementary Fig. 1F).

To investigate the prognostic capabilities of our 177 gene signature, we first measured the average expression of all 177 genes for each patient across all cancer types in the TCGA data [31] and showed statistically significantly higher expression in tumour versus normal tissue across 14 out 22 cancer types, in particular, cancers of epithelial cell origin (Supplementary Fig. 1G). Hence, our refined 177 gene metastatic signature can be detected in bulk RNA-seq datasets and holds discriminatory power for tumour versus normal samples.

Next, we calculated a metastatic score across all patients in the TCGA pan-cancer cohort and stratified patients into high or low metastatic potential using the median score within each cancer type as a cut-off (Fig. 1E). Subsequently, we modelled the relapse free survival (RFS) with high and low metastatic scores for a range of cancers. Here, we evaluated data from various established resources, including the Breast Invasive Carcinoma Collection (BRCA), head and neck squamous cell carcinoma (HNSC), Kidney Renal Clear Cell Carcinoma (KIRC), Rectum Adenocarcinoma (READ), Prostate Adenocarcinoma (PRAD) and Lung Adenocarcinoma (LUAD). In all cases, we found a high metastatic score associated with a reduced RFS (cox hazard ratio: 2.65; *p* = 7.18 × 10 − 5) (Fig. 1F). The significant classification of patients into high and low RFS across multiple cancer types supports the prognostic utility of our 177 gene signature beyond specificity to a single of these cancer types.

### Identification of molecularly distinct early disseminating cells through metastatic scoring

**Fig. 2:**
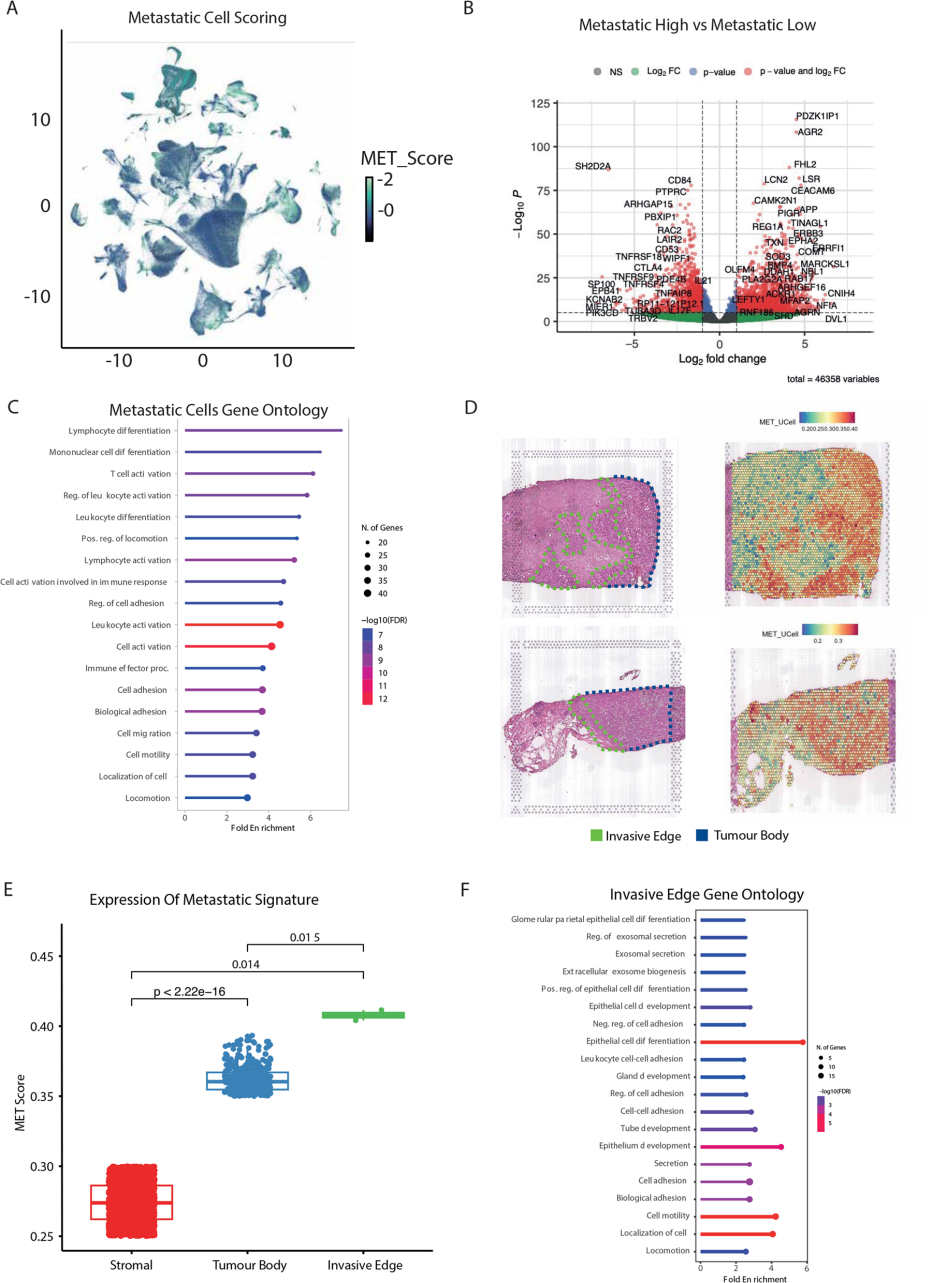
A refined metastatic signature uncovers the pan-cancer molecular landscape of metastatic cells. **A** UMAP projection of cells scored for metastatic potential from low to high using UCell. **B** Differential gene expression (DEG) analysis comparing cells with low versus high metastatic scores. **C** Gene Ontology (GO) terms associated with genes exhibiting higher expression in high metastatic potential cells. **D** Spatial transcriptomics plots illustrating tumour regions (indicated by black dashed lines) with metastatic scoring based on a 177-gene signature using UCell. (**E**) Expression patterns of the metastatic signature across stromal, tumour body, and invasive edge regions in spatial transcriptomics data. **F** GO terms enriched in genes with significantly higher expression in invasive edge clusters compared to the tumour body

To delineate the potential core mechanisms of metastasis shared across cancers, we examined our scRNA-seq data to identify cells with low and high metastatic potential. Towards this, we used the refined gene signature to score each cell from low to high metastatic potential [32]. Interestingly, most cells displayed metastatic scores in the intermediate range rather than high or low scores (Fig. 2A). Subsequently, we binarized the cell scoring into 16 distinct bins. We categorized the cells with scores at the top 20% as high, the bottom 20% as low, and the remaining 60% as mid. Using these data, we investigated the differences in the transcriptional landscape between low and high metastatic cells using pseudobulk differential gene expression analysis (Fig. 2B). Here, the genes that were significantly higher expressed in metastatic cells included many genes previously known to be involved in this process in separate cancers, including LCN2 [33, 34] and AGR2 [35] (Fig. 2B). These observations further validate our approach that the 177-core gene signature can identify cells with a high metastatic potential and further reveals a wider gene regulatory network driving this progression. Interestingly, cells with a high metastatic score showed enrichment for metastasis associated biological processes, including cell motility, locomotion, and immune activation (Fig. 2C).

Having identified cells in scRNA-seq data, we next sought to explore the utility of our signature in spatially locating metastatic cells within a tumour using spatial transcriptomics data. This approach aimed to reveal insights into potential cell–cell interactions within the tumour microenvironment and validate that our signature was selecting cells with a high metastatic potential due to their spatial locations. To this end, we obtained and analyzed the spatial transcriptomics data of four breast cancer patients containing the primary tumour, the invasive edge and the surrounding stroma [36]. By implementing UCell scoring [32] of our 177 gene metastatic signature, we successfully identified groups of cells with a high metastatic potential on each patient slide (Fig. 2D). Of note, our signature genes were most highly expressed along the invasive edge, followed by the tumour body as compared to the surrounding stroma (Fig. 2E). Furthermore, cells with a high metastatic score on the invasive edge were significantly enriched for GO terms associated with metastasis and cell migration (Fig. 2F). We further validated these observations by analyzing additional spatial transcriptomic datasets from patients with invasive breast cancer [36] and prostate cancer [37]. Specifically, we applied UCell scoring to our refined 177-gene metastatic signature and overlaid these scores onto the spatial transcriptomics plots of the respective tissues. Visually, the regions annotated as invasive carcinoma exhibited higher UCell scores, indicating elevated expression of our gene signature (Supplementary Fig. 2A-B).

To quantify this observation, we extracted the metadata from both datasets and performed a Wilcoxon rank-sum test to compare the UCell scores between invasive carcinoma regions and all other tissue regions. The statistical analysis revealed that invasive carcinoma regions had significantly higher scores than non-carcinoma regions (Supplementary Fig. 2C-D). This result reinforces the specificity and relevance of our 177-gene signature in identifying regions of invasive carcinoma within spatial transcriptomic data. Thus, our 177 gene signature can capture cells with metastatic phenotypes in both scRNA-seq and spatial transcriptomic data from different cancers. Furthermore, using our 177 gene signature, we are classifying cells that are potentially on the verge of dissemination, highlighting the validity and relevance of our signature to the pan-cancer phenomenon.

### Shared metastatic fate driven by correlated gene expression program across distinct cell types

**Fig. 3:**
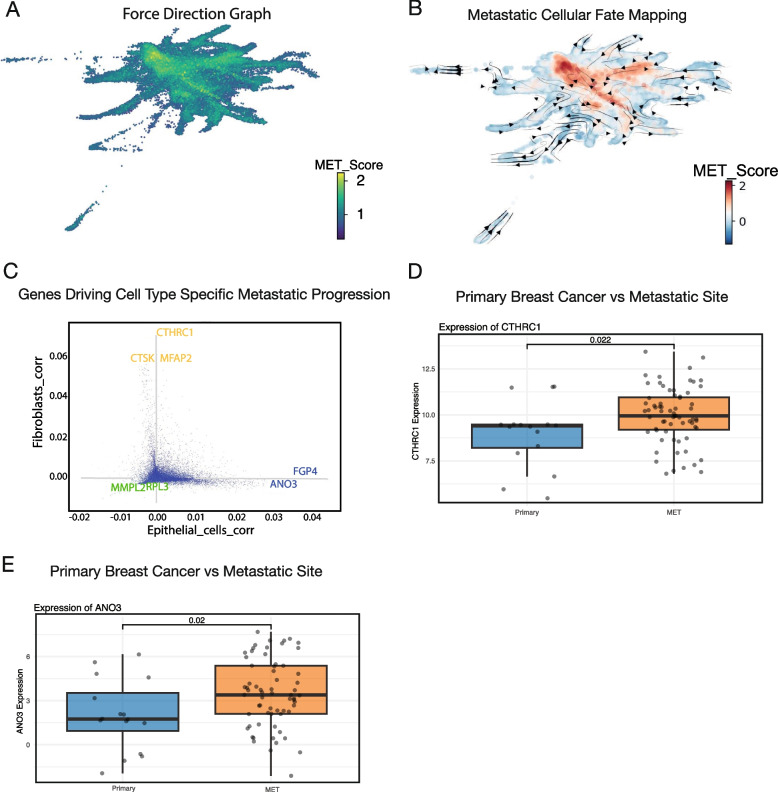
Simulated cell fate mapping reveals metastatic cellular dynamics from low to high metastatic potential across cancers. (**A**) Force-directed graph of pan-cancer single-cell RNA-seq (scRNA-seq) data, with cells coloured based on their metastatic scores. (**B**) Metastatic transition matrix illustrating cellular dynamics transitioning from low to high metastatic potential. (**C**) Identification of genes driving cell fate progression in epithelial (blue) and fibroblast (yellow) lineages during the transition from low to high metastatic potential. (**D**) Expression levels of CTHRC1 and ANO3 in primary breast cancer compared to metastatic sites, as measured by RNA-sequencing (RNA-seq)

Subsequently, to enhance the visualisation of scored cells in the scRNA-seq data, we employed force-directed graph (FDG), which depicts cellular relationships based on their mutual interactions and similarities in gene expression profiles. This analysis revealed a compelling observation in which cells with elevated metastatic scores clustered together (Fig. 3A). This spatial organisation of metastatic scored cells suggested a coherent progression or trajectory associated with metastatic scoring (Fig. 3A). To resolve the metastatic trajectories at the pan-cancer level, we used a CellRank computed KNN graph as well as the CytoTRACE pseudotime [38] (Supplementary Fig. 3A). Additionally, we calculated the directed transition matrix and transition streams on the FDG embedding to determine cellular differentiation kinetics.

Interestingly, we identified a collective metastatic trajectory from low to high metastatic potential irrespective of cancer and cell types (Fig. 3B). Importantly, CellRank revealed that cell type-specific genes drive metastasis, implying that distinct genes can drive the same metastatic fate in different cell types (Fig. 3C). Of note, a set of these genes have previously been implicated in similar processes. For example, we identified CTHRC1 as a key gene in driving metastatic progression, and it has been previously implicated in several metastatic cancers [39, 40]. In particular, CTHRC1 was shown to be secreted from cancer-associated fibroblasts in breast cancer and promote invasion, EMT processes and activation of the Wnt/β-catenin pathway [41]. In epithelial cells, ANO3 and FGP4 were identified as key drivers of metastasis and may represent novel therapeutic targets across cancers. Next, we explored the expression of our cell type-specific metastatic genes in RNA-seq data from primary breast cancer and metastatic sites [42]. CTHRC1 and ANO3 were shown to be highly expressed in metastatic tumours compared to primary tumours (Fig. 3D-E). To gain further insights into the underlying transcriptional landscape driving each distinct cell type of metastatic progression, we extracted the top genes driving this progression as calculated by CellRank for epithelial and fibroblast cells and performed gene ontology analysis on each. This showed enrichment for many cancer and metastatic related KEGG terms associated with driving this progression (Supplementary Fig. 3B-C). Of note, WNT signalling was found to be the top pathway driving epithelial metastatic progression as well as in fibroblasts, hinting that many cell types, whilst having distinct gene expression profiles, may share common signalling pathways to drive a metastatic progression.

These results highlight that correlated expression of distinct genes in specific cell types and common signalling pathways may contribute to metastatic progression across different cancers, opening the opportunity to identify novel pan-cancer biomarkers.

### Transcriptional networks underlying metastatic continuum across diverse cancer types

**Fig. 4:**
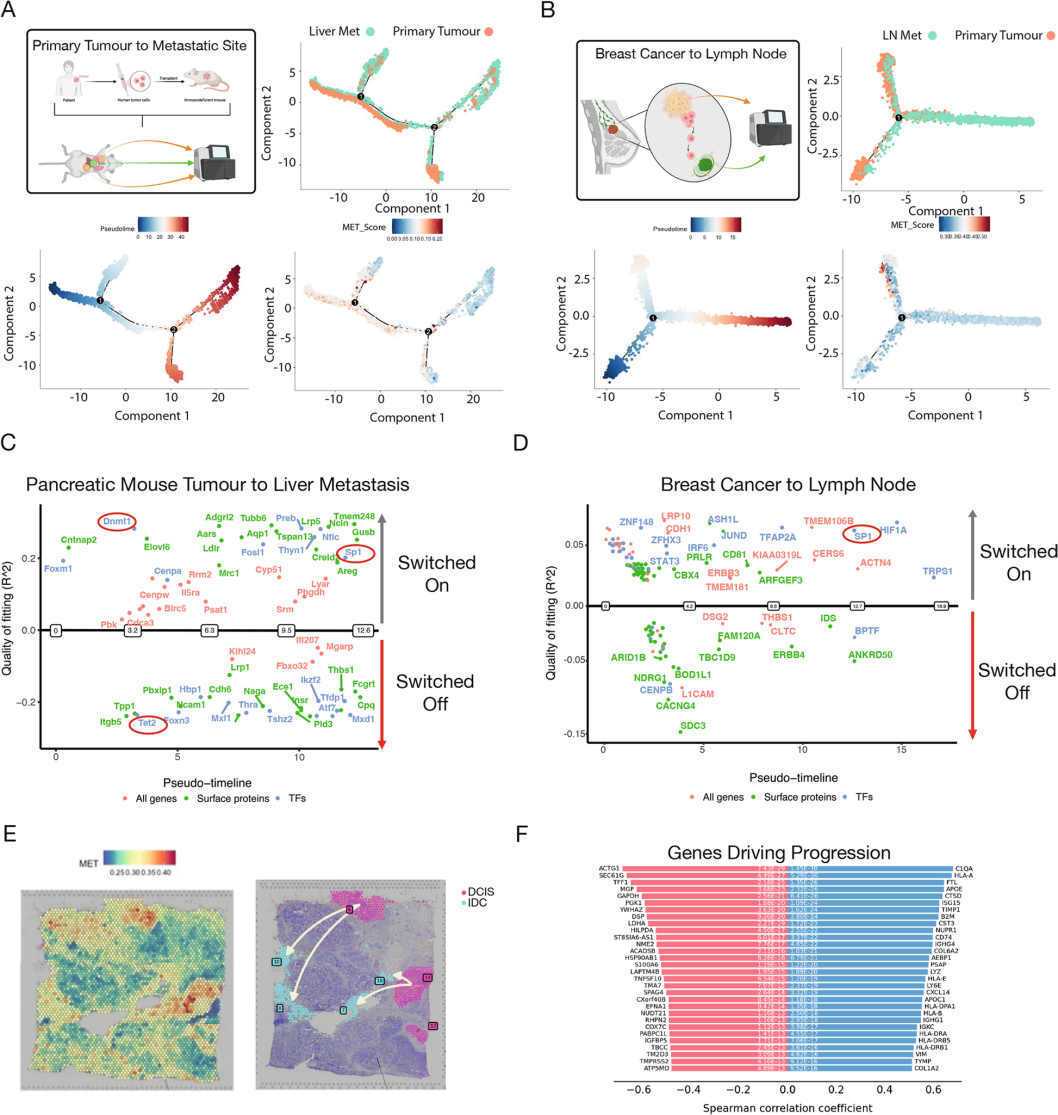
Refined metastatic signature can recapitulate the cascade of tumour migration. **A** Monocle 2 trajectory analysis of paired patient-derived xenograft (PDX) pancreatic ductal adenocarcinoma (PDAC) and liver metastasis cells, with cells coloured by site, pseudotime, and metastatic score using UCell. **B** Monocle 2 trajectory analysis of paired breast cancer and lymph node metastasis cells, similarly, coloured by site, pseudotime, and metastatic score using UCell. **C** Differential gene expression along pseudotime for the PDX PDAC and liver metastasis pair, quality of fitting is calculated using McFadden's Pseudo R^2^ **D** Differential gene expression along pseudotime for the breast cancer and lymph node metastasis pair, quality of fitting is calculated using McFadden's Pseudo R^2^ (**E**) Spatial transcriptomic profiling of a breast cancer patient, scored for metastatic potential using UCell. **F** Trajectory analysis of the breast cancer spatial transcriptomics data, highlighting genes that drive the observed cellular trajectories

Our previous analysis was based solely on primary tumour data and metastatic scoring. Next, we aimed to leverage our 177-gene metastatic signature with pseudotime analysis to identify cells within the primary tumour that have a high potential to metastasize. Additionally, we aimed to identify cells within the secondary site that may have originated from the primary tumour. To achieve this, we obtained scRNA-seq of paired primary PDAC and secondary liver tumours in a PDX model [43]. Following pre-processing, we focused our analysis on epithelial cells only. Next, using our 177 gene metastatic signature, we scored each cell using an unbiased cellular fate trajectory inference using Monocle 2 and observed a continuum. In the mouse data from the primary tumour to the liver metastatic site, the highest-scoring cells appear to align with the low and intermediate pseudotime range, while in human primary breast cancer to lymph node metastasis, they align with the intermediate and later range of pseudotime cells in a separate branch (Fig. 4A-B). Identical results were obtained using datasets from paired samples of primary PDAC to lung metastatic site in a PDX model. (Supplementary Fig. 4A&B). Furthermore, similar analyses were obtained using scRNA-seq from primary breast cancer and paired lymph node and lung metastases [44] where our 177 gene metastatic score identified cells on a continuum with the highest scoring cells spanning the intermediate and later pseudotime ranges (Fig. 4B, Supplementary Fig. 4C). These observations suggest that our 177-gene signature can identify cells undergoing dissemination and those recently metastasized to the secondary site.

We next attempted to identify the order in which genes are either switched on or off along the discovered metastasis trajectory and therefore exported our Monocle 2 objects as inputs into the R package GeneSwitches that uses a statistical framework based on logistic regression for this purpose [45]. Interestingly, this analysis in PDX primary-to-liver and human breast cancer-to-lymph node trajectory identified several distinct and shared genes that are dynamically switched on or off as cells moved towards a highly metastatic fate (Fig. 4C-D). For example, the transcription factor SP1, a known driver of metastasis, was identified to gain in activity in the later stages in both cases. In PDX primary to liver metastasis trajectory, we also found a dramatic switch between Tet2 and Dnmt1 at the earlier time points (Fig. 4C), two enzymes with opposing functions in DNA methylation, which is known to be aberrant in cancer [46].

Utilising human breast cancer spatial-transcriptomics data, we next sought to explore the trajectory from non-invasive ductal carcinoma in situ (DCIS) to invasive carcinoma and identify drivers common with our metastatic gene list. We began by using stlearn [47] to reconstruct cell type evolution in spatial transcriptomics data [47]. After scoring each region with our 177 gene metastatic signature, we aimed to reconstruct the spatial trajectory between a DCIS cluster with a low metastatic score and an IDC cluster with a high metastatic score (Fig. 4E-F). Interestingly, this analysis revealed that many genes driving this trajectory were within our pan-cancer 177 gene metastatic signature (Fig. 4F), including COL1A2, whose high expression is known to promote the tumour cell proliferation and metastasis in oesophageal cancer [48], highlighting the utility of our pan-cancer 177 gene signature in distinct cancer types. Altogether, our pan-cancer 177 gene metastatic signature can accurately identify and arrange cells along a metastatic continuum across multiple cancer types. Furthermore, these results further highlight that many of the genes within our signature potentially contribute to the dissemination of tumour cells.

### Emergence of intercellular WNT signalling from the microenvironment to tumour epithelial cells during metastatic progression

**Fig. 5:**
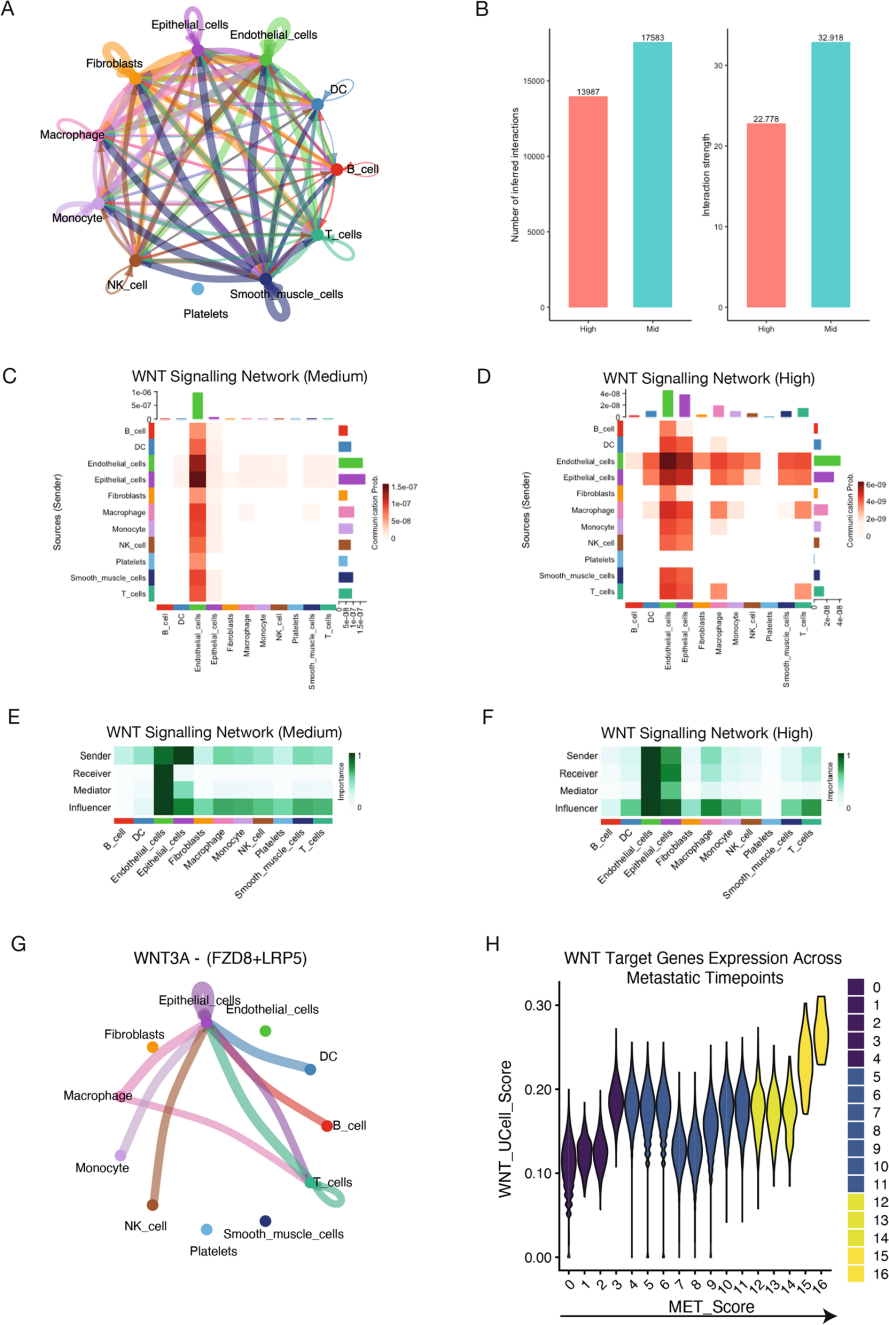
Identification of WNT signalling as a key driver of communication networks in metastatic cells. **A** Total cell–cell interactions across different cell types in samples with high metastatic potential. **B** Comparison of the number of interactions between metastatic high and metastatic medium scored cells. **C** WNT signalling interactions among cell types in metastatic medium scored cells. **D** WNT signalling interactions among cell types in metastatic high scored cells. **E** Communication networks mediated by WNT signalling in metastatic medium scored cells. **F** Communication networks mediated by WNT signalling in metastatic high scored cells. **G** Top ligand interactions driving WNT signalling in metastatic high cells. (H) UCell scoring of WNT target genes across metastatic timepoints, highlighting gradual increases in expression and the implication of WNT signalling in metastatic cells

Communication between cancer cells and the surrounding stromal cells impacts tumour proliferation, metastasis and treatment failure [49, 50]; and its disruption holds the potential to combat metastatic progression [27]. Since our 177 gene signature showed high expression in cell types beyond epithelial cells, and each distinct subtype shared similar signalling pathways (Fig. 1D), we next sought to investigate the cell-to-cell communication networks driving metastatic progression at the pan-cancer level. In the first instance, we used CellChat [51] to uncover signalling interactions between low, mid and high scored metastatic cells. We calculated the cell–cell communication network for all cell types in high metastasis scored cells, where the width of the edges represents the strength of the communication and revealed a wide range of communication networks between all cell types (Fig. 5A). Finally, we compared all the signalling pathways in cells scored as high to low (Supplementary Fig. 5A) and mid (Fig. 5B, Supplementary Fig. 5B).

Interestingly, we found that WNT signalling was most active in high metastatic cells, followed by mid and inactive in low metastatic cells across all cancers. WNT signalling has previously been implicated in metastatic progression, and it appeared among one of the top pathways driving epithelial and fibroblast metastatic cell fate (Supplementary Fig. 3A-B). However, the cell types involved in the communication networks were previously unknown [52]. In mid scored cells, endothelial cells were the dominant cell type receiving, sending, mediating (controlling signalling flow), and influencing (a hybrid measure of controlling communication) WNT signalling. In high scored cells, multiple cell types received WNT signalling, with epithelial cells emerging as the dominant receiver along with the endothelial cells (Fig. 5 C-F). Notably, we found that this communication was primarily driven by the WNT3A ligand, the FZD8 receptor and the LRP5 co-receptor (Fig. 5G). In line with these observations, LRP5 has been shown to correlate significantly with tumour metastasis [53]. The robustness of these observations was further confirmed using another highly cited tool, LIgand-receptor ANalysis framework (LIANA [54]) where we see a higher activity of WNT5A and FZD4 ligand and receptors only in metastatic cells (Supplementary Fig. 5E). Interestingly, genes encoding some of these ligand-receptor pairs were found to be bound by SP1, suggesting its potential involvement in regulating their expression cells undergoing metastasis **(**Supplementary Fig. 5F**)**.

To corroborate these findings, we performed cell–cell communication analysis on spatial transcriptomics data of invasive breast cancer using stlearn [47]. We scored cells using our 177 gene signature and calculated the communication networks between highly scored (i.e. “metastasis high”) cells and the surrounding stromal cells (Fig. 4E-F). To further substantiate these findings, we next investigated the dynamics in the expression of WNT signalling target genes during metastatic progression. Towards this, we scored each cell using UCell for WNT target gene expression and plotted their scores across each metastasis timepoint (Fig. 5H). Notably, this analysis revealed that the WNT target genes had increasing expression across metastasis time points, with high scored cells having the highest expression. This aims to validate our findings and supports previous observations that WNT signalling plays a key role in the maintenance and proliferation of tumour cells [39, 55]. Altogether, these observations establish that emergent WNT signalling from the stroma to tumour epithelial cells potentially plays a key role in the metastatic transition.

### Drug repurposing analysis reveals distinct FDA-approved drugs targeting pan-cancer metastasis

**Fig. 6:**
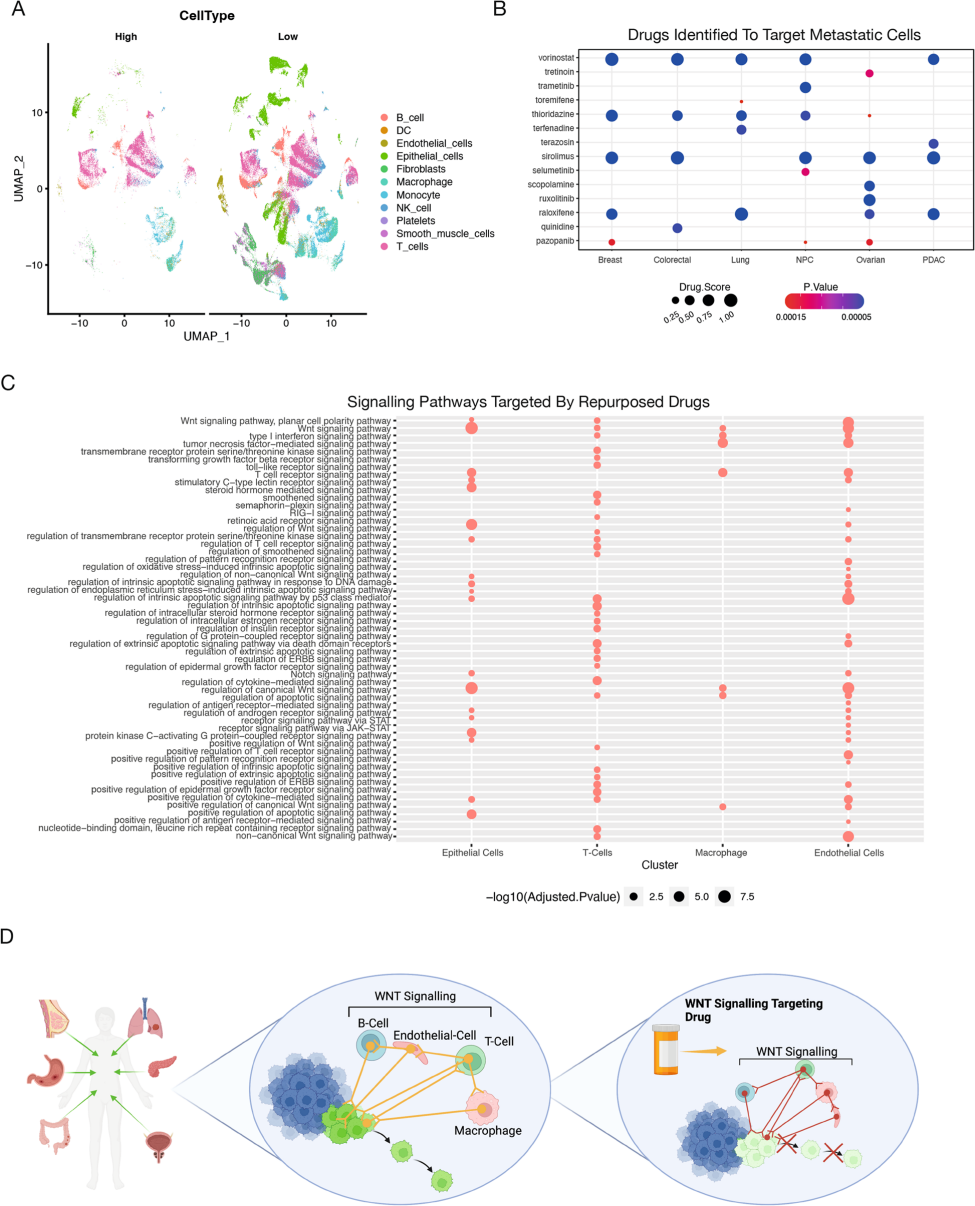
In silico drug repurposing analysis reveals FDA-approved drugs targeting metastatic cells. **A** UMAP projection of low and high metastatic cohorts, coloured by distinct cell types. **B** Drug repurposing strategy aimed at targeting metastatic cells. **C** Gene Ontology (GO) term analysis of cell type–specific genes targeted by the top three FDA-approved drugs. **D** Schematic illustrating the targeting of WNT signalling to disrupt cell–cell communication networks that drive metastatic progression

Drug repurposing has recently become highly attractive as it permits new uses of a drug outside the scope of its original medical approval or investigation, accelerating patient support [56]. Given the strong potency of our signature in identifying metastatic cells across cancers, we implemented the drug repurposing recommendation tool ASGARD [58] on our scRNA-seq data to identify any existing drugs with the potential to target metastasis. Here, we first divided our merged pan-cancer scRNA-seq dataset into low metastatic cells (bottom 20% score) and high metastatic cells (top 20% score) (Fig. 6A). We then used limma [59] to identify differentially expressed genes between cell types classified as metastasis low vs metastasis high. These consistently differentially expressed genes were then used as inputs to identify drugs that can significantly (FDR < 0.05) reverse their expression levels in the L1000 drug response dataset, which comprises 591,697 drug/compound treatments. We next applied ASGARD and predicted 15 drugs (FDR < 0.05 and overall drug score > 0.99) for the treatment of metastasis from breast, colorectal, lung, NPC, ovarian and/or PDAC cancers (Fig. 6B).

The top candidate in our repurposing analysis, Vorinostat, was shown to have a high drug score across five out of 6 cancer types. It is a histone deacetylase (HDAC) inhibitor approved for the treatment of cutaneous T-cell lymphoma and has been used to treat metastatic tumours in several cancer types and is the focus of many clinical trials, including for breast cancer treatment [60–63]. Furthermore, Vorinostat has been shown to have a potential role in modulating cell proliferation via WNT signalling and the cell cycle through degradation of β-catenin, resulting in an inhibition of cell proliferation, with a cell cycle arrest occurring in G1/G0. Additionally, Vorinostat treatment has been shown to impede cell migration [64, 65]. We performed a GO analysis to identify the target genes and pathways of the top three candidates (Vorinostat, thioridazine, sirolimus) across the top four cell type clusters based on cell type proportion in each condition (Fig. 6C). This analysis uncovered WNT signalling as a target, particularly in epithelial, endothelial, and T cells (Fig. 6C). Altogether, these findings suggest that FDA-approved drugs that disrupt WNT signalling could be repurposed to overcome or prevent metastatic progression across multiple cancer types.

### Conserved gene regulatory networks driving metastatic cascade

**Fig. 7:**
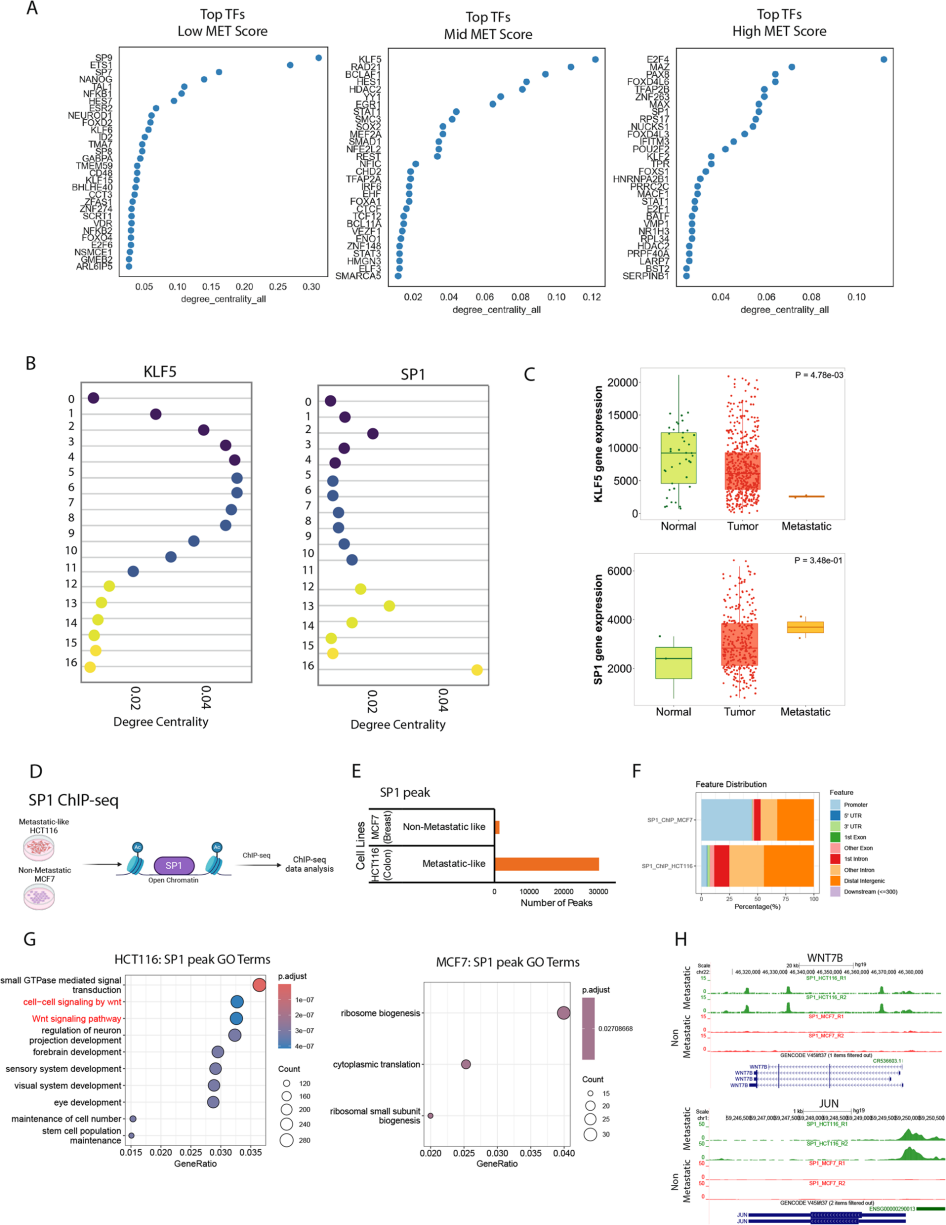
Reconstructing low to high metastatic regulatory networks conserved across cancers. **A** Transcription factors (TFs) within the gene regulatory network (GRN) associated with different metastatic stages. **B** Network dynamics of TFs across metastatic stages, coloured by MET_Score stage. **C** Expression levels of KLF5 and SP1 in normal, tumour, and metastatic samples, as measured by RNA sequencing (RNA-seq). **D** Genome browser tracks of SP1 ChIP-seq data, highlighting WNT target genes bound by SP1. **D** Schematic overview of the SP1 ChIP-seq data analysis. The SP1 ChIP-seq data were derived from ENCODE database for metastatic-like HCT116 and non-metastatic-like MCF7 cells and analysed using standard ChIP-seq analysis pipeline. **E** The barplot shows number of peaks detected for SP1 bound regions in HCT116 and MCF7 cells. **F** The barplot shows annotation of SP1 bound genes at promoter and non-promoter regions of the genome. **G** The dotplot represents top enriched pathways of SP1 bound genes in HCT116 and MCF7 cells. The top enriched pathways were derived using hallmark gene signatures from Molecular Signatures Database (MSigDB). H) The browser tracks show SP1 binding signal at hallmark WNT pathway genes

Next, we investigated how transcription factor (TF) networks contribute to the stage-specific metastasis programs we identified from scRNA-seq data. Utilising CellOracle [66] we defined gene regulatory networks, in which CellOracle [66] constructed GRN models between a TF and its target genes for each metastatic timepoint. We then utilised CellOracle [66] again to assess the contribution of each TF using centrality metrics, resulting in a final list of TFs for each metastatic score category (Fig. 7A). Here, degree centrality scoring in the high metastatic cluster gene regulatory network configuration successfully recognised key TFs associated with metastatic disease, such as SP1 and E2F4 (Fig. 7A). In contrast, the low and mid metastatic score clusters identified TFs that are known to be associated with reducing or inhibiting metastatic progression, such as STAT1 and KLF5 (Fig. 7A) [67, 68].

As we had details on each regulon across each metastatic stage, we analysed how network connectivity changes during metastasis to gain an insight into the contribution of each GRN along our metastatic continuum. We were particularly interested in two TFs, SP1 and KLF5, given their previous implications in cancer metastasis and opposite kinetics in our data during metastasis progression (Fig. 7B). Interestingly, the network scores for SP1 recapitulate the metastatic progression scores, with reduced activity in the low-to-mid scored cells and a sharp increase in the high scored cells (Fig. 7B). In contrast, KLF5 had a high network score in the mid scored cells and a sharp decrease in cells with a high metastatic score (Fig. 7B) [67].

To further validate these observations, we investigate the expression dynamics of these TFs in normal tissues, primary and metastatic tumours. We found that KLF5 had lower expression in metastatic tumours versus primary tumours and normal tissue, hence potentially acting as a tumour/metastatic suppressor, whereas SP1 displayed the opposite pattern, functioning likely as a tumour/metastatic promoter (Fig. 7C). These expression patterns clearly highlight that our observed continuum correlates with metastatic cancer progression and is also in line with previous observations where high expression of SP1 was found to be associated with an unfavourable prognosis across multiple cancer types, which directly correlates with TNM staging [69].

We next sought to explore the possibility of SP1 in directly regulating WNT-related genes in metastatic cells. Towards this, we processed SP1 ChIP-seq from a metastatic (Colon: HCT116) and a non-metastatic (Breast: MCF7) cancer cell line from the ENCODE database and further analysed to identify SP1-drive transcriptional circuitry in these cell models (Fig. 7D). Interestingly, we found a dramatically larger number of regions bound by SP1 in metastatic cells compared to non-metastatic cells, suggesting a more active gene regulatory role of SP1 in metastatic cells (Fig. 7E). Notably, SP1 binding occurs more in the distal regulatory regions within metastatic cells compared to non-metastatic cells, where SP1 occupancy is at the proximal regulatory sites (Fig. 7F). Given the established critical role of distal regulatory elements in defining cell identity, SP1 potentially functions as a dominant driver of metastatic cell features in these cells. Notably, these data showed a clear occupancy of SP1 at regulatory elements of several WNT target genes, such as WNT7B and JUN genes in metastatic cells, but did not target these loci in non-metastatic like cells [70–73] (Fig. 7G-H). These results suggest that SP1 is an upstream inducer of WNT signalling genes during metastatic progression. Overall, our data uncovered key TFs that function at different stages of metastatic progression to drive essential driver pathways.

### SP1 controls cell survival, invasive growth and metastatic colonisation

**Fig. 8:**
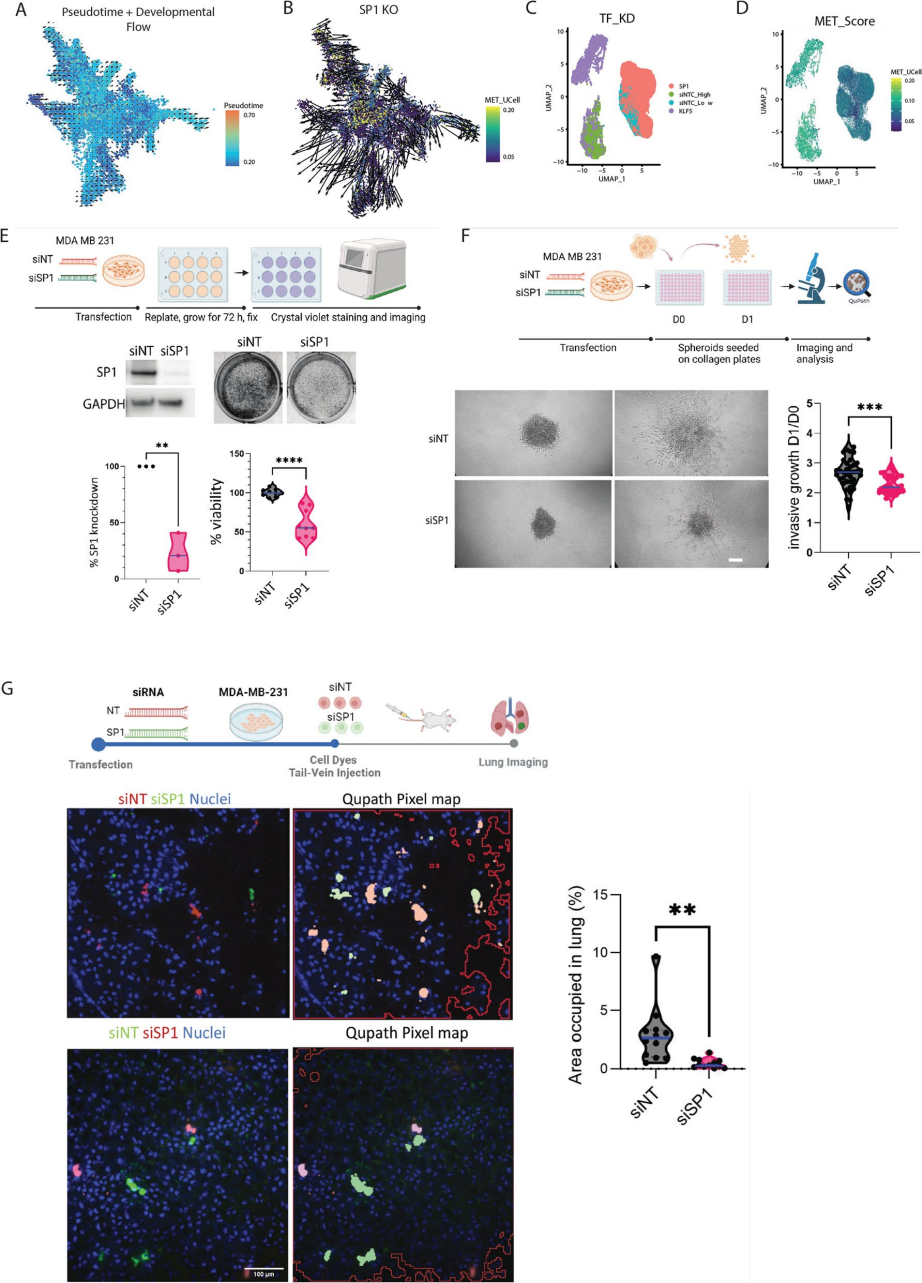
SP1 and KLF5 have opposing roles in the metastasis program. **A** Pseudotime calculation of cells, showing overlap with metastatic scoring and the transition from low to high metastatic potential cells. **B** In silico perturbation of SP1 alters the metastatic transition trajectory. **C** UMAP projections of MB231 (high metastatic) and HCC1806 (low metastatic) cells, coloured by transcription factor knockdown (TF KD) and non-targeting control (siNTC) cohorts. **D** UMAP projection with cells scored from low to high metastatic potential using UCell. **E** Representative immunoblotting images of SP1 knockdown and non-targeting control (NT) MDA-MB-231 cells (*n* = 4). **F** Representative Crystal Violet assay images for viability in SP1 knockdown and non-targeting control (NT) MDA-MB-231 cells (*n* = 4), with quantification of absorbance at 595 nm (right). **G** Representative images and quantitation of knockdown and control MDA-MB-231 spheroid growth embedded in Collagen-I at day 0 (D0) and day 1 (D1), scale bar = 50 μm (*n* = 3). **H** Schematic of lung colonisation assay using vital dye-stained SP1 knockdown (red) and control MDA-MB-231 cells (green) co-injected into the tail vein of NXG mice. Lungs were imaged 24 h post-injection, displaying representative images with a heatmap analysis using QuPath pixel mapping. Cell nuclei are stained with Hoechst 33,342. Quantitation of the area occupied by fluorescent cells in the lungs (%) for siNT and siSP1 MDA-MB-231 cells is shown (right). Scale bar = 100 μm. The same experiment was repeated with inverted vital dye colours (SP1 knockdown in green and NT controls in red) (*n* = 2). Violin plots display median (blue) with interquartile ranges. *p*-values were calculated using unpaired t-tests. All n numbers indicate independent experiments unless otherwise stated

As a proof-of-principle for prioritising TFs as novel therapeutic targets, we examined our breast cancer scRNA-seq and perturbed SP1 in silico (Supplementary Fig. 6A). We scored each cell using our 177 gene signature on an FDG layout of the breast cancer subset and again found that metastatic cells appeared to cluster together (Supplementary Fig. 6B). We first calculated the pseudotime and development flow using CellOracle [66] and found it followed the sample progression as our metastatic score (Fig. 8A). Next, we used CellOracle-based simulation of SP1 perturbation to recapitulate the progression from low to high metastatic cell fate. Using the 16 metastatic gene regulatory network configurations inferred by CellOracle, we simulated SP1 knockout signal propagation (expression set to 0 across all cells), enabling the prediction of future gene expression, and hence the direction of cell identity transitions, at single-cell resolution. This simulation predicts a visual shift of high metastatic cell identity toward a low metastatic signature following SP1 knockout (Fig. 8B).

We repeated this analysis in a mouse scRNA-seq dataset containing primary and matched lung metastases to validate these findings. We scored each cell using our 177 gene signature and calculated the gene regulatory networks at each metastatic stage, which revealed Sp1 as a key regulator in highly metastatic cells (Supplementary Fig. 6C-D). Interestingly, another transcription factor, Klf2, was found to have the highest activity in mid scored cells(Supplementary Fig. 6D) and is a well-known repressor of metastasis in multiple cancer types [74, 75]. Calculating the pseudotime and development flow using CellOracle followed the sample progression as our metastatic score (Supplementary Fig. 6E). Notably, the *in-silico* perturbation of SP1 predicted a clear shift from high to low metastatic potential (Supplementary Fig. 6F). Targeting SP1 TFs has been previously shown to disrupt metastatic cancer *in vitro* [76]*.* Collectively, these findings suggest that SP1 inhibitors could potentially prevent or reverse metastatic progression across multiple cancer types.

Following the initial in silico analysis, we sought to validate and gain deeper insights into the transcriptional and cell-fate changes resulting from SP1 and KLF5 knockdown in vitro. For this purpose, we identified the MDA-MB-231 cell line as having a high metastatic potential and the HCC1806 cell line as having a low metastatic potential based on our 177-gene signature (Supplementary Fig. 7A-B). Subsequently, we conducted SP1 depletion in the highly metastatic MDA-MB-231 cell line and KLF5 knockdown in the low metastatic potential HCC1806 cell line and performed single-cell transcriptome (scRNA-seq) analysis in biological replicates. Further analysis revealed that the loss of SP1 made the highly metastatic cells cluster together with the low metastatic potential control cells (Fig. 8C). Moreover, SP1 knockdown cells exhibited a significant reversal in metastatic scoring of the 177 genes, confirming the inhibitory effects of SP1 knockdown (Fig. 8D). Furthermore, gene ontology analysis revealed a remarkable decrease in the expression of genes associated with metastasis (Supplementary Fig. 7C-D). For example, key pathways related to GTPase signalling were prominently downregulated, indicating a potential suppression of the metastatic phenotype [77, 78]. Additionally, genes associated with cell migration exhibited reduced expression levels, further supporting the role of SP1 in governing the metastatic gene expression program. Overall, these results highlight a significant role of SP1 in driving the gene expression program underlying metastatic progression.

Conversely, KLF5 knockdown in HCC1806 cells showed a significant increase in metastatic scoring, resulting in KLF5 knockdown cells clustering with the high metastatic potential control cells and separating from the control, poorly metastatic HCC1806 cells, indicating an increase in metastatic potential following KLF5 knockdown (Fig. 8C-D). In direct contrast to the SP1 depletion, the gene ontologies for KLF5 loss were enriched for pro-metastatic biological processes (Supplementary 7Fig E–F). For example, GTPase signalling pathways and genes associated with cell migration were significantly upregulated, confirming an enhancement in metastatic potential (Supplementary Fig. 8E).

To further confirm our in-silico findings, we performed SP1 knockdown in MDA-MB-231 cells and subjected them to a set of assays that measure features of metastatic cells. First, we observed reduced cell viability compared to control cells (Fig. 8E-F). Next, we measured the invasive growth of SP1 knockdown cells using the 3D spheroid assay on collagen [79]. We observed reduced invasive growth in SP1 knockdown cells compared to control (Fig. 8F). Finally, we performed in vivo lung colonisation assays that measure cancer cell survival in the lung parenchyma. Briefly, siRNA-transfected cells stained with vital dyes were co-injected at a 1:1 ratio into the tail-vein of immunocompromised mice, and the percentage of cells retained in the lungs was quantified [80–82]. We observed that SP1 knockdown cells were less competent to grow in the lung parenchyma than control cells (Fig. 8G). Overall, this data suggests that SP1 controls several features of metastatic cancer cells in vitro and in vivo.

### SP1-driven WNT Pathway activity is essential for metastatic features

**Fig. 9:**
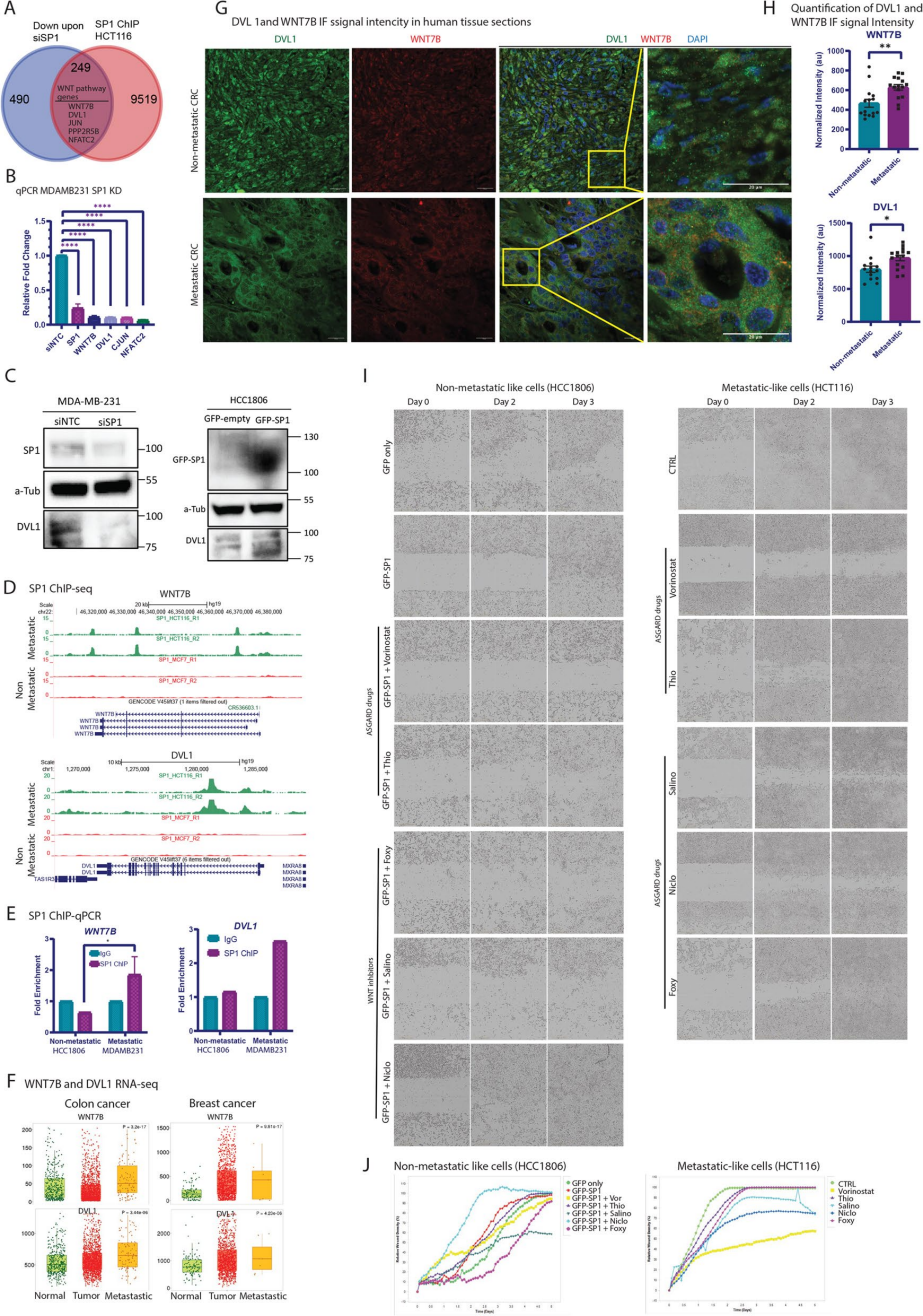
Induction of WNT pathway genes by SP1 drives metastatic features. **A** Venn diagram shows overlap of SP1 bound genes with downregulated genes upon siSP1. The five genes shown inside the venn diagram are WNT pathway genes. **B** The bar graph shows relative qPCR fold change for the expression of GAPDH, SP1, WNT7B, DVL1, JUNC and NFATC2 upon SP1 knockdown in MDA-MB-231 cells. Each bar indicates the mean of replicate values. Error bar indicates SEM. For statistical analysis, student ‘s t-test is performed (* *p* < 0.05, *** *p* < 0.001). Gene expression was normalized to the corresponding expression of each gene upon siNTC control knockdown in MDA-MB-231. **C** Immunoblotting for SP1 knockdown in MDA-MB-231 (left plot) cells against SP1, a-Tubulin and DVL1. A-Tubulin served as a loading control. siNTC: siRNA for non-targeting control. The right plot showing Immunoblotting for GFP-SP1 overexpression in HCC1806 cells against GFP, a-Tubulin and DVL1. A-Tubulin served as a loading control. siNTC: siRNA for non-targeting control. **D** The browser tracks show SP1 binding signal at hallmark WNT pathway genes WNT7B and DVL1 in HCT116 and MCF7 cells. **E** ChIP-qPCR relative fold enrichment for SP1 binding at the promoter sites of WNT7B and DVL1. Each bar indicates the mean of replicate values. Error bar indicates SEM. For statistical analysis, student ‘s ttest is performed (* *p* < 0.05). IgG is used as a negative antibody control for ChIP. Non-metastatic: HCC1806 cells; metastatic: MDA-MB-231 cells. **F** The boxplot showing expression of WNT7B and DVL1 in normal, primary and metastatic tumors from colon and breast cancer patients. The expression levels were derived from TNMplot database. **G** Representative images of Immunofluorescent staining for non-metastatic and metastatic CRC human tissue section. DVL1 in green, WNT7B in red and DAPI in blue. Scale bar indicates 20 microns. Region of interest is marked in dashed yellow rectangle. H) Quantification of immunofluorescent signal intensity for WNT 7B and DVL1. Each bar represents the mean intensity. Error bars indicate SEM. Each dot represents the quantification value for each individual region. For statistical analysis, student ‘s t test was performed (* *p* < 0.05, ** *p* < 0.01). **I** Representative bright field images of incucyte experiment for SP1 overexpressing HCC180 and highly metastatic HCT116 cancer cells with and without treatment with the five selected drugs (vorinostat, Thiaridazine, Niclosamide, Salinomycin and Foxy 5 on Day 0, 2 and 3. **J** Quantification for the incucyte experiment coupled with all drug treatments shown in (I). Vor: vorinostat, Thio: thioridazine, Salino: salionomycin, Niclo: niclosamide, Foxy: Foxy-5

To explore a direct role for SP1 in driving WNT pathway activity to promote metastatic features, we further analyzed the SP1 ChIP-seq datasets from metastatic (HCT116) and non-metastatic (MCF7) cancer cells (shown in Fig. 7D-H). By overlapping SP1-bound genes with those misregulated upon SP1 knockdown, we identified a subset of targets, including key WNT pathway genes (WNT7B, DVL1, JUN, PPP2R5B, and NFATC2), that were downregulated in the absence of SP1 (Fig. 9A).

To further validate SP1's regulation of WNT pathway genes, we performed SP1 knockdown in two metastatic cancer cells MDA-MB-231 and HCT116. The results showed a marked reduction in cell migration upon SP1 knockdown compared to controls (Supplementary Figure S8C, D), consistent with the previous assays (Fig. 8E, F) that demonstrated reduced metastatic potential. Importantly, WNT7B and DVL1 were significantly downregulated following SP1 knockdown (Fig. 9B-C), reinforcing the idea that the WNT pathway is a downstream target of SP1 in regulating metastasis. We also investigated whether SP1 overexpression alone in non-metastatic cells is sufficient to induce metastatic behavior. Interestingly, such ectopic overexpression of SP1 in HCC1806 cells resulted in a dramatically faster migration, as confirmed by scratch assays and Incucyte Live-Cell imaging (Supplementary Figure S8E-F; Fig. 9I, J). Furthermore, this accompanied an induction of WNT pathway genes at the RNA (Supplementary S8A) and protein level (Fig. 9C).

ChIP-seq analysis further showed that SP1 binds at the regulatory elements of these genes only in metastatic cancer cells and not in non-metastatic cells (Fig. 9D). To independently confirm the occupancy of SP1 at WNT pathway genes, we performed chromatin immunoprecipitation assay followed by qPCR (ChIP-qPCR) for WNT7B and DVL1 loci. The results show a clear occupancy of SP1 at the regulatory elements of these genes in independent metastatic cancer cells (MDA-MB-231) but not in non-metastatic (HCC1806) cancer cells (Fig. 9E).

We extended our analysis by investigating WNT pathway activity in metastatic tumors. First, we injected metastatic breast cancer cells (MDA-MB-231) into mice and collected tumor samples post-metastasis. Staining for WNT7B and DVL1 in these tumors revealed their high expression levels and a strong co-localization (Supplementary S8B). Furthermore, RNA-seq analysis in a large cohort revealed that the expression of SP1-target WNT pathway genes were significantly elevated in metastatic tumors compared to the controls (Fig. 9F). These observations were further validated by immunohistochemistry in colorectal cancer patient samples, where DVL1 and WNT7B exhibited significantly higher and more homogeneous expression in metastatic tissues compared to non-metastatic ones (Fig. 9G, H).

Lastly, we aimed at testing whether such SP1-driven metastatic behaviour can be disrupted by pharmacological approaches for therapeutic purposes. In our earlier analysis, we had identified vorinostat and thioridazine as the top drugs targeting metastatic cells in majority of the cancer types (5 of 6 cancer types) in ASGARD analysis which ranks FDA-approved drugs against cell populations using scRNA-seq datasets (Fig. 6B). Furthermore, given the activation of WNT signaling in metastatic cells, we also shortlisted three WNT pathway inhibitors: Niclosamide and Salinomycin (both FDA-approved), and Foxy-5 (currently in Phase 2 clinical trials). Interestingly, application of these drugs in two independent highly metastatic cancer cell lines, MDA-MB-231 (breast) and HCT116 (colon), severely impaired their migratory capacity to varying degrees, with some cell-type specific exceptions (Fig. 9I, J; Supplementary Figure S8 G-I). Furthermore, similar effects were observed for SP1-overexpression induced migratory behaviour in normally non-metastatic cancer cells (HCC1806) (Fig. 9I, J). These findings underscore the essential role of the SP1/WNT axis in metastasis and highlight the potential for therapeutic targeting of this pathway to inhibit cancer progression.

## Discussion

Our study presents a comprehensive analysis of a 177-gene signature that offers significant insights into the metastatic progression of various cancer types. This pan-cancer approach underscores the utility of a unified genetic framework to understand the complex biology underlying metastasis across diverse tumour types and microenvironments. The 177-gene signature highlights key molecular pathways involved in metastatic dissemination, including those related to cell adhesion, migration, and extracellular matrix remodelling. Notably, several genes within this signature have previously been implicated in metastatic processes in specific cancers, suggesting a broader applicability of these molecular mechanisms across multiple cancer types.

The results demonstrate that this gene signature can serve as a predictive tool for metastatic potential, providing a valuable resource for both basic and translational cancer research. The ability to predict metastasis using a common genetic signature could facilitate earlier intervention and personalised treatment strategies, potentially improving patient outcomes. Moreover, the integration of this gene signature with existing clinical parameters could enhance the accuracy of prognostic models. Future studies should aim to validate these findings in larger, independent cohorts and explore the therapeutic implications of targeting these key pathways. Our analysis reveals that the metastatic scoring across samples follows a continuous distribution, highlighting the nuanced spectrum of metastatic potential within individual cells. Utilising cellular dynamics modelling on single cells, we observed that cells with lower metastatic scores tend to progress towards a higher metastatic state. This trend is consistent across various cell and cancer types, suggesting a universal trajectory towards metastasis, which presents novel opportunities for therapeutic intervention aimed at halting metastatic progression universally across cancers. Interestingly, while this shared metastatic fate is common among different cell and cancer types, each exhibits a unique underlying transcriptional program. Notably, genes such as ANO3 and CTHRC1 emerged as novel contributors to pan-cancer metastasis, with their expression levels showing a direct correlation. This finding underscores the potential for these genes to serve as biomarkers or therapeutic targets, warranting further investigation.

Moreover, our exploration into the transcriptional programs driving cellular progression towards metastatic high scored cells identified WNT signalling as the primary pathway influencing metastatic progression. This pathway's significant role across different cell types underscores its potential as a target for broad-spectrum anti-metastatic therapies. WNT signalling has been reported to be involved in metastatic progression [55, 83]. Our findings reveal specific WNT signaling networks involved in cell-to-cell communication at the single cell level during metastatic progression. This insight opens the opportunity to target WNT signalling to disrupt these communication networks and prevent metastatic progression.

We further show that a higher WNT activity is primarily governed by the SP1 transcription factor in high metastatic cells. High levels of SP1 protein have been shown to correlate with tumour cell migration and metastasis in a number of tumour models and patient samples, including gastric and breast cancers [84–88] and WNT signalling activity [89, 90]. However, a role for SP1-WNT signalling axis in metastatic progression had remained poorly understood, thereby highlighting the importance of our study which has clearly filled this knowledge gap that applies at the pan-cancer level. Furthermore, our *in-silico* knockout in breast cancer and paired primary and metastatic site mouse scRNA-seq datasets showed that a loss of SP1 in high metastatic cells could reverse the fates to a low metastatic state.

Through our detailed mechanistic investigation in vitro and in vivo validation, we confirmed that SP1 knockdown in metastatic cells MDA-MB-231 and HCT116 resulted in significant suppression of metastatic features which accompanied a downregulation of WNT pathway genes such as WNT7B, DVL1, and JUN. Noticeably, these genes were strongly bound by SP1 in metastatic cells but not in non-metastatic cells. These observations were further confirmed in human tissue sections as well as RNA-seq of low and high metastatic tumours, hence demonstrating the clinical relevance of these findings. Conversely, overexpression of SP1 in lowly metastatic cancer cells was sufficient to confer a migratory behaviour which accompanied upregulation of these markers both at the RNA and protein levels.

Furthermore, clustering analysis confirmed the inhibitory effects of SP1 knockdown, while KLF5 knockdown in low metastatic HCC1806 cells resulted in the opposite effect, enhancing metastatic gene ontologies and upregulating GTPase signalling. GTPase signalling plays a crucial role in cancer metastasis. GTPases, such as Ras and Rho, act as molecular switches that regulate cellular processes, including migration [91]. Activation of GTPases promotes dynamic changes in the cytoskeleton, leading to cell protrusions and motility, and Rho GTPases control actomyosin contractility [92]. Dysregulation of GTPase signalling contributes to enhanced cancer cell migration and invasion, promoting metastatic dissemination [93]. Understanding the intricate interplay between GTPase signalling pathways and cell migration mechanisms is vital for developing targeted therapies to impede cancer metastasis and improve patient outcomes. These intriguing findings shed light on the complex and context-dependent roles of SP1 and KLF5 in cancer metastasis. Of note, there have previously been links between KLF5 expression and SP1 in breast and prostate cancers, linking elevated KLF expression with tumour suppressive functions [94]. Further experiments showed that SP1 is critical for cancer cell survival, invasive growth and metastatic colonisation, highlighting its critical importance in metastasis at the pan-cancer level. In conclusion, our integrated analysis of scRNA-seq and metastatic scoring data reveals the opposing roles of SP1 and KLF5 in regulating metastasis by governing the underlying gene expression program in cancer cells. Ultimately, these findings contribute to a better understanding of the metastatic process and offer potential targets for precision medicine approaches in cancer treatment. Future studies aimed at unravelling the downstream targets and crosstalk between SP1 and KLF5 could provide valuable insights into novel therapeutic strategies for managing metastatic breast cancer.

Lastly, we set out to explore whether our findings can be used for pharmacological targeting to block metastatic activity across cancers. Our ASGARD analysis revealed FDA-approved vorinostat and thiaridazine as the most effective drugs in targeting metastatic cells in the majority of cancer types. Additionally, given our findings on the activation of WNT signaling in metastatic cells, we included three WNT pathway inhibitors: Niclosamide and Salinomycin (both FDA-approved), and Foxy-5 (in Phase 2 clinical trials). Notably, applying these drugs to naturally metastatic cancer cells or cancer cells forced to migrate with SP1 overexpression markedly reduced their migratory abilities. Vorinostat is a histone deacetylase (HDAC) inhibitor, FDA-approved for the treatment of cutaneous T cell lymphoma [95, 96]. In addition, several preclinical and clinical investigations have its promising potential in inhibiting metastasis in cancer [97–100]. Thiaridazine is a low-potency typical antipsychotic but has been shown to have anticancer effects in the brain [101, 102] and aggressive breast cancer [103, 104]. Similarly, WNT inhibitors salinomycin, Niclosamide and Foxy-5 have previously been known to have anti-tumour activity across cancers [105–109]. These findings demonstrate the therapeutic potential of these drugs in targeting and preventing metastasis in aggressive cancers.

## Conclusions

In this study, we investigated pan-cancer core mechanisms of metastasis by performing the largest single-cell transcriptome analysis involving over 200 patients with both metastatic and non-metastatic tumours across six cancer types. Our findings uncovered a prognostic core gene signature that sheds light on the complex cellular dynamics and gene regulatory networks that govern metastasis. Specifically, the examination of transcription factor networks active at different stages of metastasis, along with functional perturbation experiments, identified SP1 and KLF5 as key regulators at critical transition steps of this process. SP1 acts as a driver of metastasis, while KLF5 functions as a suppressor across multiple cancer types at defined time points of the metastatic transition. In vivo and in vitro loss-of-function studies of SP1 in cancer cells demonstrated its critical role in promoting cancer cell survival, invasive growth, and metastatic colonisation. Moreover, our results showed that as metastasis advances, tumour cells and their microenvironment increasingly communicate through WNT signalling, a process that is driven by SP1. Supporting these findings, a drug repurposing analysis identified several FDA-approved drugs with potential anti-metastasis properties, including inhibitors of WNT signalling, effective across various cancer types. These findings mark a groundbreaking advancement in cancer research by unveiling the core gene regulatory circuitry driving metastasis conserved across various cancers and discovering novel therapeutic avenues.

## Methods

### Processing and annotation of scRNA-seq

Single-cell RNA sequencing data obtained through droplet-based 10 × Genomics technology was selectively utilised for meaningful comparisons in the analysis. Raw count matrices consisting of Unique Molecular Identifiers (UMIs) and corresponding cell metadata were aggregated from diverse sources (Supplementary Table 1).

### Quality control

Preliminary quality control procedures were conducted for each individual dataset using the Seurat R package (v4.0). Cells selected for subsequent analysis were those with more than 200 detected genes and genes identified in a minimum of 3 cells. To identify and eliminate potential cell doublets, scDblFinder v1.6.0 was applied with default parameters. Cells manifesting a mitochondrial transcript content exceeding 20% were also excluded to mitigate confounding effects. To identify samples with fewer than 100 malignant cells we utilised inferCNV with default parameters.

### Normalisation and transformation

Normalisation was achieved by scaling UMI counts with respect to library size, followed by a log transformation to stabilise variance. Subsequently, the dataset underwent Principal Components Analysis (PCA) to reduce dimensionality.

### Clustering and visualisation

Utilising the first 30 principal components, a nearest neighbour graph was constructed, facilitating subsequent clustering via the Louvain algorithm at a lower resolution (FindClusters, resolution = 0.2). For illustrative purposes, uniform manifold approximation and projection (UMAP) embeddings were produced based on 30 principal components, enabling effective visualisation.

### Cell Identity annotation

SingleR v1.6.1 in conjunction with the Human Primary Cell Atlas from the celldex R package (v1.2.0) was employed to accurately annotate individual cell identities within each sample. Importantly, the references for annotation encompassed expected cell types within the tumour microenvironment (TME), which include, but are not limited to, B cells, dendritic cells, endothelial cells, epithelial cells, fibroblasts, macrophages, monocytes, NK cells, platelets, smooth muscle cells, and T cells.

### Multiresolution archetypal analysis

A multiresolution archetypal analysis was performed on each cancer cell derived from a total of 222 tumour samples. This process was accomplished using the ACTIONet software (v2.1.7). The primary goal was to disentangle the gene expression profiles of the cells into a concise collection of latent expression programmes. These programmes exhibited heterogeneous expression patterns across the cellular population.

### Kernel matrix reduction

The initial step involved the computation of reduced kernel matrices. This was achieved using the "reduce.ace()" function, a feature of the ACTIONet package in R. The parameter "reduced_dim = 20" was specified for this operation.

### Selective ACTIONet parameters

As each population exclusively represented a distinct cell type, specific parameters were employed for running ACTIONet. Specifically, the "k_max = 10" option was set to ensure a controlled reduction. To mitigate the influence of a small number of cells on archetype generation, the parameter "min_cells_per_arch = 5" was used. This safeguarded against the emergence of archetypal expression programmes driven by only a handful of cells.

### Pseudotime analysis

The Monocle2 package (v2.8.0) was used to analyse single-cell trajectories to discover the cell-state transitions from GSE180286 and GSE173958. We used the top 100 differentially expressed genes in CNV cluster cells identified by Seurat to sort cells in pseudo-time order. ‘DDRTree’ was applied to reduce dimensions and the visualisation functions ‘plot_cell_trajectory’ were used to plot the minimum spanning tree on cells. we get three states of cancer cells. Next, RNA-velocities were predicted using scVelo in the python program.

### GeneSwitches analysis

To determine the significant genes that regulate the trajectory from primary to pre-metastatic cells, GeneSwitches v0.1.0R package was applied. Cells from the primary tumour site were first extracted from the specific trajectories. Then the corresponding single-cell log-transformed gene expression and monocle pseudo-time were input into GeneSwitches. Function *binarize_exp* with fixed cut-off 0.2 was used to binarise the gene expression into on or off states. For each gene, *find_switch_logistic_fastglm* function calculated a switching time and associated confident level. Top 50 genes of high confident levels, including surface proteins and transcription factors, were plotted using function *plot_timeline_ggplot* to visualise the switching orders.

### Gene set scoring

Gene set scoring was performed using the R package UCell v1.0.0. UCell scores are based on the Mann–Whitney *U* statistic, which evaluates the rank of each query genes’ expression level in individual cells. Because it is rank-based, the scores are independent of the cellular composition of the dataset and are interpretable as the relative ranking of the gene set within the cell’s transcriptome.

### Pan-cancer TCGA pre-processing

The bulk RNA-seq profiles and clinical outcome data were downloaded from the online repository at https://gdc.cancer.gov/node/905. For each sample, RNA-seq count data underwent normalisation to counts per million, followed by a logarithmic transformation to achieve stability in the data.

###  Pan-cancer TCGA metastatic signature calculation

To quantify metastatic signature scores, the average gene-level Z score of cancer cell-specific genes was computed for each sample. This calculation provided a measure of metastatic signature activity within each sample.

### Metastatic signature association with clinical outcomes

We assessed the relationship between metastatic signature activity and progression-free interval (PFI) using a Cox proportional hazards model. This model incorporated tumour type, purity, stage, and age as covariates, along with continuous metastatic scores. These analyses aimed to uncover potential associations between metastatic signature activity and clinical outcomes. To address the issue of multiple comparisons, *P* values were adjusted using the Benjamini–Hochberg method. This correction ensured robustness in our statistical inferences while considering the possibility of chance findings.

### Spatial transcriptomics analysis

The UMI count matrix underwent processing using the R package Seurat (v4.0) with default parameters. Initially, the data were normalised using sctransform, and principal component analysis was conducted to reduce the dimensionality of the log-transformed gene-barcode matrices of top variable genes. To pinpoint molecular features correlating with spatial location within a tissue, Seurat was employed for differential expression based on pre-annotated anatomical regions within the tissue. Subsequently, the metastatic score for each ST spot was calculated using UCell.

### Spatial co-localisation of receptor-ligand pairs

To investigate cell–cell interactions between DCIS and ICS, we identified significant ligand-receptor pairs between neighbouring spots using CellPhoneDB, as implemented in StLearn.

### Spatial trajectory analysis

To explore cellular trajectories in situ, we reprocessed the raw spatial data following documented protocols in StLearn. Post-Louvain clustering and global and local pseudo-space–time trajectory analyses were carried out in StLearn. Genes showing differential upregulation or downregulation along the trajectories were identified using Spearman’s rank correlation, with a set threshold of 0.3.

### Cellular dynamics of metastatic cells

CytoTRACE (v.0.3.3) and CellRank (v.1.5.1) were used to infer the metastatic trajectory of tumour and TME cells using CellRank tutorials. Following the creation of the log_2_-normalised expression matrix, the predicted orders were projected onto the pan-cancer force direction graph. CellRank was performed to map the cell fate of metastatic cells from low to high metastatic potential.

### Cell–cell communication analysis

To evaluate cellular interactions between different high- and low-metastatic cells, we applied CellChat (v.1.1.3) to infer ligand-receptor interactions from the scRNA-seq data. We used the normalised count data as an input and followed the CellChat tutorial with default parameters and CellChatDB.human as the interaction database. Cellular interactions were visualized using the netVisual_circle function. The cellchat based cellular communication analysis was further validated using another widely used software LIgand-receptor ANalysis framework (LIAN) tool (PMID: 35,680,885) which is a LIgand-receptor ANalysis frAmework as an open-source interface to all the resources and methods. We ran the LIANA using consensus mode with filtered interactions specific to WNT signaling pathway.

### ChIP-seq data analysis

SP1 ChIP-seq dataset of metastatic-like HCT116 cells and non-metastatic cells MCF7 was obtained from ENCODE database, HCT116: ENCSR000BSF and MCF7: ENCSR729LGA, reads were aligned to HG19 using bowtie and peaks were called using MACS2 and resulting files were visualised using UCSC genome browser. The peaks were further annotated to identify SP1 occupancy in these cell lines using annotatePeak() in ChIPseeker R package. Pathway enrichment analysis of SP1 bound genes was performed enrichGO() in clusterProfiler R package.

### CellOracle GRN analysis

We built gene regulatory networks (GRNs) with CellOracle (v.10.10) using default parameters.

### In silico perturbation

In silico perturbation was performed using CellOracle as previously defined using default parameters with SP1 expression set to 0.

### ASGARD Drug repurposing screen

We used the normalised count data as an input, with metastatic low-scoring cells set as the control, and followed the ASGARD tutorial with default parameters. We calculated individual drug scores for each cancer type to identify drugs with a significant score across each.

### Cell culture

The TNBC line HCC1806 was maintained in RPMI 1640 (Gibco, 21,875,034) medium supplemented with 10% FBS, 1% glucose, and 1 mM sodium pyruvate (Thermo, 11,360,070). MDA-MB-231 cells were maintained in DMEM (Dulbecco’s modified Eagle’s medium) with 10% FBS. Cells were grown as monolayers at 37 °C in a humidified CO2 (5%) incubator.

### siRNA transfection

The scrambled siRNA control and ON-TARGETplus SMARTpool siRNA targeting human SP1 and KLF5 were purchased from Dharmacon. Transfection was performed using Lipofectamine™ RNAiMAX (Invitrogen, 13,778,150) according to the manufacturer’s instructions. In brief, cells were seeded at 225 k/well for MDA-MB-231. Cells were seeded at 250 k/well for the HCC1806 cell line. All cells were seeded the day before the transfection. siRNA at a final concentration of 20 pmol was diluted in 125 μL of Opti-MEM (Gibco, 31,985,047) and 7 μl of Lipofectamine RNAiMAX was diluted in 125 μl of OPTI-MEM. The diluted siRNA and Lipofectamine RNAiMAX were mixed and incubated at room temperature for 10 min. 250 ul of the transfection mixture were added to each well of the six-well plates. Twenty-four hours later, the transfection cocktail was replaced with complete media for each cell line.

### Single-cell RNA-seq library preparation

Cells were collected at 200 × g for 5 min, fixed using the Parse Biosciences Cell Fixation Kit (ECF2001) and stored at −80 °C. To create a single-cell suspension, cells were resuspended in the Cell Fixation Additive Buffer provided by the Parse Cell Fixation Kit. Then the RNase inhibitor contained prefixation buffer, which was added for the next fixation. Cell pellets were collected after 10 min of centrifugation at 200 × g at 4 °C. The cell suspension containing BSA (Thermo Fisher 15,260,037) was strained through a 40 µm strainer (Corning 431,750) to achieve a single-cell suspension. Cell numbers were then counted. Samples were preserved at −80 °C until the initiation of bar-coding and library prep with the Evercode Whole Transcriptome kit (ECW02030).

### SP1 and KLF5 knockdown scRNA-seq data analysis

After sequencing, the FASTQ files representing each sub-library were demultiplexed into individual FASTQ files, with each file representing one single cell using the PARSE pipeline (*v1.1.0*). Sequencing reads were mapped to the human transcriptome (HG38) using the PARSE pipeline (*v1.1.0*). Output files were then loaded into Seurat using the function ReadParseBio(). Downstream analysis, including normalisation and dimension reduction, was performed as above.

### ChIP-seq data analysis

SP1 ChIP-seq data was obtained from ENCODE, ID: ENCSR000BSF, reads were aligned to HG19 using bowtie and peaks were called using MACS2 and resulting files were visualised using UCSC genome browser.

### Cell lines

In vitro viability and invasion assays, and in vivo lung colonisation assays were carried out in the triple negative breast cancer MDA MB 231 cell line. All experiments were repeated at least three times.

### Transfections

For siRNA based knockdowns, reverse transfections in MDA MB 231 cells were carried out in 6 well plates. 7.5 μL of Lipofectamine 2000 (Invitrogen) was mixed with 250 μL of OptiMEM media (Gibco) while 4 μL of OnTarget siRNA pool (Dharmacon) was mixed in 250 μL of OptiMEM media. After 5 min, both solutions were mixed and incubated at room temperature for 20 min. 400,000 cells/well were resuspended in 2.5 mL per well of a 6 well plate and 500 μL of the solution was added to each well. The media was changed the next day and functional assays were initiated 48 h later.

### Immunoblotting

72–96 h after siRNA transfection, cells were lysed in Laemmli lysis buffer and snap frozen in −80 °C. Lysates were boiled directly at 95 °C for 10 min, sonicated for 120 s and spun at 13,000 g for 20 min. Lysates were then resolved in non-reducing SDS-PAGE gels using 4–12% gradient precast gels (NuPAGE, Invitrogen), and proteins transferred to 0.45 μm PVDF membranes (Immobilon-P) subsequently. Membranes were blocked in 4% BSA in 0.1% Tween 20-TBS for 1 h at room temperature, and probed with SP1 antibody (21,962–1-AP, ProteinTech, 1:2000) or GAPDH (MAB374, Invitrogen, 1:10,000). For chemiluminescent detection, ECL Prime detection system (Amersham) in a BioRad ChemiDoc MP machine was used.

### Spheroid assays

For invasive growth in spheroid assays, the hanging drop method (Del Duca et al., 2004) was employed to generate spheroids from 2000 cells per drop. Briefly, 40,000 cells were resuspended in 500 μL of low viscosity media (80% complete DMEM and 20% 1X MethylCellulose solution), and 25 μL were plated on the lid of a 60 mm dish for overnight hanging drop formation. The next day, spheroids were transferred to 2.2 mg/mL Purecol matrix, and allowed to invade for 24 h. The area invaded was normalised to the spheroid area on D0, and fold change in invasive growth was calculated using Qupath.

Pictures of Day 0 and Day 1 were taken at 4 × magnification using a brightfield phase contrast microscope. A QuPath project was created, and annotations from Day 0 were transferred to Day 1. Once transferred, the annotations were expanded by 100 µm. A pixel classifier was then developed to detect and differentiate areas of the tumour from the ignore class (background). This classifier was applied, allowing for the calculation of the percentage of invasion.

### Viability assays

For viability assays, control and knockdown MDA MB 231 cells were replated at a density of 100,000–200,000 cells/well in a T12 dish, 48 h after transfection. After 72 h of growth, cells were fixed with 4% PFA for 15 min, washed twice with PBS and stained with 0.5% Crystal Violet stain for an hour. Excess stain was washed off and the plates allowed to dry overnight. The stain was dissolved in 10% Acetic Acid and absorbance read at 595 nm.

### In vivo lung colonisation assays

For experimental metastasis assays, MDA-MB-231 cells were transfected with non-targeted control (NT) or siSMART pool SP1 (siSP1). After 48 h from transfection cells were labelled with 10 μM CMFDA-Green (C7025, Life Technologies) or 10 μM CMRA-Orange (C34551, Life Technologies) for 10 min and then trypsinised and counted. Mixed cells ratio (1:1) total number of 1 0.5 × 10^5^ labelled cells / 0.1 ml were co-injected into tail vein of 36-week-old female NOD/SCID/ IL2Rγ-/- mice (NSG, Charles River). Mice were sacrificed 24 h (to confirm that equal numbers arrived at the lung) and 24 h after tail vein injection. Lungs were extracted, washed with PBS (with calcium/magnesium) twice and fixed with 4% formaldehyde for 16 h at 4 °C and co-stained with DAPI. Lungs were examined using Super Resolution Spinning Disk (TIRF/STORM/SoRa) with C-Apochromat X 20 objective lens, CSU-W1 camera and i3 Marianas software. z-stacks used in analyses of collagen attachments, 30 μm thick stacks were taken with a fixed step size of 0.5 μm. Analysis of lung fluorescence images was performed in QuPath. An average threshold from three channels (Blue, Green, and Orange) was created. This average was then used as a general mask and created as a single annotation. Two independent thresholds for the Green and Orange channels were made using the simple threshold tool. Threshold masks for Green and Orange were generated as detection measurements inside the higher-level annotation. Data are presented as the percentage of the area covered by fluorescence, with 10 fields per mouse analysed. n = 6 mice/condition for each experiment. Experiments were done twice and each time the colours for each condition were swapped.

### Animal licence ethics

All animals were maintained under specific pathogen-free conditions and handled in accordance with the Institutional Committees on Animal Welfare of the UK Home Office (The Home Office Animals Scientific Procedures Act, 1986). Animals were housed in the QMUL Biological Services holding facility, which maintained a 7 h light/dark cycle, an ambient temperature of 19–22 °C and humidity of 50–60%. All animal experiments were approved by the Ethical Review Process Committees at Barts Cancer Institute and King’s College London, in accordance with the Animals (Scientific Procedures) Act 1986 and according to the guidelines of the Committee of the National Cancer Research Institute.

### Statistical analyses

Student’s unpaired t-test was conducted for tests of significance, using GraphPad Prism. Data were plotted as graphs as Mean ± SEM. Level of significance was denoted using *p*-values, with *p* < 0.05 considered statistically significant (*P < 0.05, **P < 0.01, ***P < 0.001, and ****P < 0.0001). Further statistical analyses were performed using R (version 4.1.1). Student's *t*-test, Wilcoxon rank-sum test and Kaplan–Meier were utilised in this study. *p*-values of less than 0.05 were considered statistically significant.

### Immunofluorescence staining on FFPE tissues

Human CRCnon-metastatic/metastatic FFPE tissue sections were purchased from the company Biochain, and MDA-MB-231-injected mouse breast xenograft breast tissue section was kindly shared by an in-house collaborator Mikkel Green Terp. Tissue sections were deparaffinized in xylene for 10 min, followed by immersion in 99% ethanol for 5 min, 96% ethanol for 3 min, and rinsed in running water for 5 min. For antigen retrieval, the sections were boiled in TEG buffer (10 mM Tris, 0.5 mM EGTA, pH of 9.0.) for 15 min, then cooled for 15 min before being rinsed with TBS. After drying gently with the tissue paper, a circle was drawn around the FFPE section with a PAP-pen. Blocking of non-specific binding was performed in 1X blocking buffer (For 1 ml: 50 ml horse serum, 250 ml 10% BSA, 700 ml 1X PBS with 0.1% Triton X-100 in PBS) for 2 h at RT. It was washed with PBS for 5 min. Primary antibodies were diluted in 0.5X blocking buffer (For 2 ml: 1 ml 1 × blocking buffer and 1 ml 1X PBS with 0.1% Triton −100). The antibodies used and dilutions applied were as follows: WNT7B, biorbyt, #orb100915, Rb, 1:100; DVL-1, Santa Cruz, #sc-8025, Ms, 1:50. Primary antibody incubation was performed at 4 °C in a humidified chamber for overnight. Then, the slides were washed three times with PBS during 3 min for each wash. Secondary antibodies were diluted in 0.5X blocking buffer (AF 488 donkey anti-mouse IgG (H + L), Thermofisher #A21202, 1:500; AF568 Donkey anti-Rabbit IgG (H + L), Thermofisher #A10042, 1:500) and incubated in a humidified chamber at RT to protect light for 30 min. Next, it was followed with 2 times PBS wash during 3 min each and 2 times ddH2O wash during 5 min each. Mounting was done in 1–2 drops of mounting medium with DAPI (Abcam #ab104139). The slides were then let to dry for 30–60 min in the dark and kept in refrigerator. The imaging was performed using confocal microscope (Nikon Eclipse Ti2-E Inverted, #52,634). Images were quantified using FIJI. In total, 15 independent regions of interest were randomly selected from 3 independent images. Mean intensity for each region of interest were measured, and the data was plotted as a scatter plot illustrating the average mean for WNT7B and DVL1 signal intensity using Graphpad prism.

### Chromatin immunoprecipitation assay

A chromatin immunoprecipitation (ChIP) assay was performed as described previously (Pataskar et al., 2016a). Briefly, MDA-MB-231 and HCC1806 cells were cross-linked in a medium containing 1% formaldehyde for 10 min at room temperature, neutralised with 125 mM glycine, scraped off and rinsed twice with 10 ml of ice-cold 1 × PBS. The cells were pelleted by centrifugation for 7 min at 4 °C at 600 g. The pellets were resuspended in 10 ml of buffer L1 [50 mM HEPES KOH (pH 7.5), 140 mM NaCl, 1 mM EDTA (pH 8.0), 10% glycerol, 5% NP-40 and 0.25% Triton X-100] and incubated at 4 °C for 10 min. This step was followed by centrifugation for 5 min at 4 °C at 1300 g. The pellet was resuspended in 10 ml of buffer L2 [200 mM NaCl, 1 mM EDTA (pH 8.0), 0.5 mM EGTA (pH 8.0) and 10 mM Tris (pH 8.0)] and incubated at room temperature for 10 min, followed by centrifugation for 5 min at 4 °C at 1300 g. The pellet was resuspended in buffer L3 [1 mM EDTA (pH 8.0), 0.5 mM EGTA (pH 8.0), 10 mM Tris (pH 8.0), 100 mM NaCl, 0.1% Na-deoxycholate and 0.17 mM N-lauroyl sarcosine] containing protease inhibitors, sonicated using a Covaris ME220 (Duration: 720, Peak power: 75, Duty factor: 20, Cycles: 400) and incubated overnight at 4 °C. After clearing the cellular debris by spinning at 14,000 g for 10 min at 4 °C, 30 μg of chromatin was incubated overnight at 4 °C with 1 µg of SP1 antibody (Proteintech, 21,962–1-AP) after 1 h of preclearing. The mixture was then incubated with 40 μl of protein A- -Agarose beads that had been preblocked with tRNA and BSA for 3 h at 4 °C. The beads were washed twice with 1 ml of buffer L3 and once with 1 ml of DOC buffer [10 mM Tris (pH 8.0), 0.25 M LiCl, 0.5% NP-40, 0.5% Na-deoxycholate and 1 mM EDTA], and the bound chromatin was eluted in 1% SDS/0.1 M NaHCO3. Next, treatment with RNase A (0.2 mg/ml) was performed for 30 min at 37 °C followed by treatment with proteinase K (50 μg/ml) for 2.5 h at 55 °C. The cross-linking was reversed at 65 °C overnight with gentle shaking. The DNA was purified by phenol–chloroform extraction followed by ethanol precipitation and was recovered in 40 μl of TE buffer.

### Quantitative RT-PCR

Total RNA of cultured cells was prepared using TRIzol reagent (Invitrogen) and reverse–transcribed with a First Strand cDNA Synthesis kit (Thermofisher). The transcripts were quantified by qPCR using Power-up SYBR Green Master Mix (Applied Biosystems) on a Lightcycler 480 II (Roche). The sequences of all primers used in this study are provided in Table Primers.

### siRNA Knockdown and transfection

SiRNA was purchased from Horizon Discovery (On target plus human SP1, 5 nmol) and prepared in a stock concentration of 20 μM. MDA-MB-231 cells (6 × 106 cells/well) were seeded overnight in 12-well plate. 0.8 μl siRNA was mixed in 45 μl Optimem (Gibco), and 2.25 μl Lipofectamine RNAimax (Thermofisher) was mixed in 45 μl Optimem. Transfection reagents were mixed and incubated for 5–10 min at RT. The cocktail was added to the cells and the cells were collected then after 2–4 days. Then the downstream experiments, including WB, qPCR and wound healing, were performed. SP1 -carrying pEGFP-C1 (Addgene) expression plasmid was transfected into the HCC1806 cells. 5 μg plasmid was mixed in 300 μl Optimem (Gibco) and 15 ul PEI-max (Polysciences), incubated for 15 min. Downstream experiments, including WB, qPCR, wound healing and incucyte, were performed after 48 h of transfection.

### Immunoblotting

The cells were lysed in RIPA lysis buffer (Millipore, 10X), and lysates from samples were boiled in 6 × SDS-PAGE loading buffer, run on a polyacrylamide gel, transferred to a PVDF membrane, blocked with 5% milk and probed with the appropriate antibodies overnight at 4 degrees. (DVL-1, Santa Cruz, #sc-8025, SP1, Proteintech, #21,962–1-AP, GAPDH, Santacruz, #sc-47724, GFP, Thermofisher, #A11122). After washing three times with TBS-T, secondary HRP-conjugated antibodies (Santacruz) were applied for 1 h at RT. After washing three times with TBS-T, the membranes were treated in Super Signal West Femto (Thermofisher) and bands were visualized using iBright 1500 device.

### Wound healing

Briefly, the MDA-MB-231, HCC1806 and HCT116 cells (6 × 106 cells/well) were plated in 12-well plates for 48 h to a confluence of 100%, then wounded by scratching with a p200 pipette tip. Thereafter, the debris was removed and washed twice with PBS. The cells were then incubated with DMEM medium containing 10% FBS and treated with 1 mM of drugs. The control sample contained the cells treated only with DMSO vehicle. For wound healing assay coupled to live imaging, incucyte (Sartorius) was used. The automated camera took images every 2 h during 5–7 days. For the manual wound healing assay, the images were taken in different time intervals by Invitrogen EVOS M300 microscope. Cell migration was assessed by FIJI, with an in-house wound healing tool macro.

### Statistical analysis

All results are expressed as means ± SEM and were obtained from three separate experiments. The results were performed with independent sample *t*-tests. The experimental and control means were compared and the differences between them were assessed for significance. A *p*-value less than 0.05 was considered statistically significant.

## Supplementary Information

Supplementary Material 1. Supplementary Figure 1. scRNA-seq of each cancer type and scoring of each patient (A) Archetypal analysis of lung and breast cancer patients, with each archetype scored using both gene lists. (B) Gene Ontology (GO) enrichment results for the 286 metastatic gene signature. (C) Stratification of cancer patients based on metastatic scoring. (D) Correlation between the 286-gene signature scores and patient stages (r = 0.134). (E) GO enrichment analysis of the 177-gene signature. (F) GO enrichment analysis of the 109-gene set. (G) Average expression levels of the refined gene signature across TCGA pan-cancer datasets, where * indicates significantly higher expression in tumours compared to normal tissues. Supplementary Figure 2. Spatial transcriptomics scoring and pseudotime analysis (A) Spatial transcriptomic map of a breast cancer patient scored for metastatic potential using a 177-gene signature with UCell. (B) Spatial transcriptomic map of a prostate cancer patient scored for metastatic potential using the same 177-gene signature with UCell. (C) Expression patterns of the metastatic signature across invasive carcinoma and other annotated regions in breast cancer spatial transcriptomic data. (D) Expression patterns of the metastatic signature across invasive carcinoma and other annotated regions in prostate cancer spatial transcriptomic data. Supplementary Figure 3. Genes driving cell type specific metastatic progression (A) Schematic overview of the CellRank method, illustrating how cells are arranged and mapped based on their cellular fate trajectories toward a common endpoint. (B) KEGG pathway enrichment analysis of the top genes driving metastatic progression in epithelial cells. (C) KEGG pathway enrichment analysis of the top genes driving metastatic progression in fibroblast cells. Supplementary Figure 4. Extended pseudotime analysis (A-C) pseudotime analysis with metastatic scoring of cells using UCell. Supplementary Figure 5. Cell-Cell communication analysis. (A) Comparison of signalling pathway–mediated cell–cell communications between high and low metastatic potential samples; (B) comparison of signalling pathway–mediated cell–cell communications between high and medium metastatic potential samples; (C) spatial transcriptomic profiling of a breast cancer patient, illustrating the spatial distribution of gene expression within the tumour microenvironment; (D) identification of top ligands driving cell–cell communications in the breast cancer spatial transcriptomics dataset; and (E) The upper heatmap shows cell-cell interaction between the cell types of high, mid and low metastatic cells. The colour intensity (light to dark) shows stronger interaction between the cell types. The bottom dot plot shows Ligand-receptors involved WNT in cell-cell communication in high, mid and low metatstatic cells. The cell-cell communication analysis was performed using LIANA package and the signalling assessed between epithelial cells and other cell types. F) the venn diagram shows overlap of the downregulated genes upon siSP1 with SP1 bound targets and WNT signaling ligand-receptors of high, mid and low metastatic datasets. Supplementary Figure 6. Cell oracle GRN inference. (A) Overview of the in-silico perturbation approach using CellOracle, illustrating the methodology for modelling gene regulatory networks. (B) Metastatic scoring of breast cancer single-cell RNA-seq (scRNA-seq) data, depicting the transition of cells from low to high metastatic potential. (C) Metastatic scoring of mouse primary tumours and paired liver metastasis scRNA-seq data, highlighting the differences in metastatic potential between primary and metastatic sites. (D) Identification of SP1 as a key regulator in highly metastatic cells and KLF2 as a regulator in medium-scored cells. (E) Pseudotime analysis showing the overlap between metastatic scoring and the transition of cells from low to high metastatic potential. (F) In silico perturbation of SP1, demonstrating its impact on altering the metastatic transition trajectory. Supplementary Figure 7. Gene Ontology Results of SP1 and KLF5 knockdown. (A) Expression levels of the metastatic signature in non-targeting control (siNTC) and SP1 knockdown (KD) MDA-MB-231 (MB231) cells; (B) Expression levels of the metastatic signature in siNTC and KLF5 KD HCC1806 cells; (C) Gene Ontology (GO) enrichment analysis of genes upregulated in MB231 siNTC cells; (D) GO enrichment analysis of genes upregulated in MB231 SP1 KD cells; (E) GO enrichment analysis of genes upregulated in HCC1806 siNTC cells; and (F) GO enrichment analysis of genes upregulated in HCC1806 KLF5 KD cells. (G) Expression of WNT target genes in SP1 KD and siNTC High cell line (H) ASGARD results on SP1 KD and siNTC High cell lines. Supplementary Figure 8. (A) Relative qPCR fold change for the expression of GAPDH, SP1, WNT7B, DVL1, JUNC and NFATC2 upon SP1 overexpression in HCC1806 cells. Each bar indicates the mean of replicate values. Error bar indicates SEM. For statistical analysis, student‘s t-test is performed (* *p* < 0.05, *** *p* < 0.001). Gene expression was normalized to the corresponding expression of each gene upon GFP only overexpression in HCC1806 cells. B) Representative images of Immunofluorescent staining for MDA-MB-231-injected mouse breast tumour tissue section. DVL1 in green, WNT7B in red and DAPI in blue. Scale bar indicates 20 microns. C) Representative bright field images of wound healing assay using MDA-MB-231 cells treated with control (siNTC) and SP1 siRNA (siSP1) after 0, 6 and 20 hours. The dashed lines in blue marks the initial wound area at 0h. Scale bar indicates 200 microns. D) Quantification of the relative wound area (%) for the wound healing assay in part A. Each bar represents the mean of triplicate values. Error bars indicate SEM. For statistical analysis, student‘s t-test was performed (* *p* < 0.05). E). Representative bright field images of wound healing assay using HCC1806 cells transfected with control (GFP-empty) and SP1-overexpressing plasmid (GFP-SP1) after 0, 2, 16 and 24 hours. The dashed lines in blue marks the initial wound area at 0h. Scale bar indicates 200 microns. F) Quantification of the relative wound area (%) for the wound healing assay in part A. Each bar represents the mean of triplicate values. Error bars indicate SEM. For statistical analysis, student‘s t-test was performed (* *p*< 0.05). G) Quantifications for wound healing assays following various drug treatments in MDAMB231 cells. H) Images of crystal violet-staining for wound healing assay using HCT116 and MDA-MB-231 cells treated with increasing doses of drugs (0, 0.2 uM, 1 uM and 4 uM). C: control (DMSO), V: vorinostat, T: thioridazine, S: salionmycin, N: niclosamide, F: Foxy-5.

Supplementary Material 2. Sheet1: scRNA-seq datasets used in this study. Sheet2: 177 metastatic genes. Sheet3: 109 metastatic genes. Sheet4: CellRank gene lists driving Epithelial and Fibroblast cell fate. Sheet5: Primer sequences used for cloning, qPCR and ChIP-qPCR.

## Data Availability

No datasets were generated or analysed during the current study.

## Abbreviations

scRNA-seq: Single-cell transcriptome profiling
FDA: Food and Drug Administration
TF: Transcription Factor
PDX: Patient derived xenograft
TCGA: The Cancer Genome Atlas Program
KD: Knockdown
DCIS: Ductal carcinoma in situ
TME: Tumor Microenvironment
UMI: Unique Molecular Identifiers
WNT: Wingless/Integrated
UMAP: Uniform Manifold Approximation and Projection

## References

[1] Parker AL, Benguigui M, Fornetti J, Goddard E, Lucotti S, Insua-Rodríguez J, et al. Current challenges in metastasis research and future innovation for clinical translation. Clin Exp Metastasis. 2022;39:263–77. 35072851 10.1007/s10585-021-10144-5 PMC8971179

[2] Broggio J, Bannister N. Cancer survival by stage at diagnosis for England. Newport, UK Off Natl Stat 2016.

[3] Chitty JL, Filipe EC, Lucas MC, Herrmann D, Cox TR, Timpson P. Recent advances in understanding the complexities of metastasis. F1000Research 2018; 7: 1169. 10.12688/f1000research.15064.1 PMC6073095 30135716

[4] Fares J, Fares MY, Khachfe HH, Salhab HA, Fares Y. Molecular principles of metastasis: a hallmark of cancer revisited. Signal Transduct Target Ther. 2020;5:28. 32296047 10.1038/s41392-020-0134-x PMC7067809

[5] Welch DR, Hurst DR. Defining the Hallmarks of Metastasis. Cancer Res. 2019;79:3011–27. 31053634 10.1158/0008-5472.CAN-19-0458 PMC6571042

[6] Ye X, Weinberg RA. Epithelial-Mesenchymal Plasticity: A Central Regulator of Cancer Progression. Trends Cell Biol. 2015;25:675–86. 26437589 10.1016/j.tcb.2015.07.012 PMC4628843

[7] Micalizzi DS, Maheswaran S, Haber DA. A conduit to metastasis: circulating tumor cell biology. Genes Dev. 2017;31:1827–40. 29051388 10.1101/gad.305805.117 PMC5695084

[8] Qian C-N, Mei Y, Zhang J. Cancer metastasis: issues and challenges. Chin J Cancer. 2017;36:38. 28372569 10.1186/s40880-017-0206-7 PMC5379757

[9] Lambert AW, Pattabiraman DR, Weinberg RA. Emerging Biological Principles of Metastasis. Cell. 2017;168:670–91. 28187288 10.1016/j.cell.2016.11.037 PMC5308465

[10] Eccles SA, Welch DR. Metastasis: recent discoveries and novel treatment strategies. Lancet. 2007;369:1742–57. 17512859 10.1016/S0140-6736(07)60781-8 PMC2214903

[11] Fedele P, Ciccarese M, Surico G, Cinieri S. An update on first line therapies for metastatic breast cancer. Expert Opin Pharmacother. 2018;19:243–52. 29336185 10.1080/14656566.2018.1425680

[12] Karagiannis GS, Pastoriza JM, Wang Y, Harney AS, Entenberg D, Pignatelli J et al. Neoadjuvant chemotherapy induces breast cancer metastasis through a TMEM-mediated mechanism. Sci Transl Med 2017; 9. 10.1126/scitranslmed.aan0026. 10.1126/scitranslmed.aan0026 PMC5592784 28679654

[13] D’Alterio C, Scala S, Sozzi G, Roz L, Bertolini G. Paradoxical effects of chemotherapy on tumor relapse and metastasis promotion. Semin Cancer Biol. 2020;60:351–61. 31454672 10.1016/j.semcancer.2019.08.019

[14] Bernards R, Weinberg RA. Metastasis genes: A progression puzzle. Nature. 2002;418:823–823. 12192390 10.1038/418823a

[15] Riggi N, Aguet M, Stamenkovic I. Cancer Metastasis: A Reappraisal of Its Underlying Mechanisms and Their Relevance to Treatment. Annu Rev Pathol Mech Dis. 2018;13:117–40. 10.1146/annurev-pathol-020117-044127 29068753

[16] van ’t Veer LJ, Dai H, van de Vijver MJ, He YD, Hart AA, Bernards R et al. Expression profiling predicts outcome in breast cancer. Breast Cancer Res 2002; 5: 57. 10.1186/bcr562 PMC154139 12559048

[17] van ’t Veer LJ, Dai H, van de Vijver MJ, He YD, Hart AAM, Mao M et al. Gene expression profiling predicts clinical outcome of breast cancer. Nature 2002; 415: 530–536. 10.1038/415530a 11823860

[18] Chang HY, Sneddon JB, Alizadeh AA, Sood R, West RB, Montgomery K, et al. Gene Expression Signature of Fibroblast Serum Response Predicts Human Cancer Progression: Similarities between Tumors and Wounds. PLoS Biol. 2004;2: e7. 14737219 10.1371/journal.pbio.0020007 PMC314300

[19] Kikuchi T, Daigo Y, Katagiri T, Tsunoda T, Okada K, Kakiuchi S, et al. Expression profiles of non-small cell lung cancers on cDNA microarrays: Identification of genes for prediction of lymph-node metastasis and sensitivity to anti-cancer drugs. Oncogene. 2003;22:2192–205. 12687021 10.1038/sj.onc.1206288

[20] Schell MJ, Yang M, Missiaglia E, Delorenzi M, Soneson C, Yue B, et al. A Composite Gene Expression Signature Optimizes Prediction of Colorectal Cancer Metastasis and Outcome. Clin Cancer Res. 2016;22:734–45. 26446941 10.1158/1078-0432.CCR-15-0143 PMC4802496

[21] Klein EA, Haddad Z, Yousefi K, Lam LLC, Wang Q, Choeurng V, et al. Decipher Genomic Classifier Measured on Prostate Biopsy Predicts Metastasis Risk. Urology. 2016;90:148–52. 26809071 10.1016/j.urology.2016.01.012

[22] Fan C, Oh DS, Wessels L, Weigelt B, Nuyten DSA, Nobel AB, et al. Concordance among Gene-Expression–Based Predictors for Breast Cancer. N Engl J Med. 2006;355:560–9. 16899776 10.1056/NEJMoa052933

[23] Priestley P, Baber J, Lolkema MP, Steeghs N, de Bruijn E, Shale C, et al. Pan-cancer whole-genome analyses of metastatic solid tumours. Nature. 2019;575:210–6. 31645765 10.1038/s41586-019-1689-y PMC6872491

[24] Zhang Y, Chen F, Creighton CJ. Pan-cancer molecular subtypes of metastasis reveal distinct and evolving transcriptional programs. Cell Reports Med. 2023;4: 100932. 10.1016/j.xcrm.2023.100932 PMC9975284 36731467

[25] Nguyen B, Fong C, Luthra A, Smith SA, DiNatale RG, Nandakumar S, et al. Genomic characterization of metastatic patterns from prospective clinical sequencing of 25,000 patients. Cell. 2022;185:563-575.e11. 35120664 10.1016/j.cell.2022.01.003 PMC9147702

[26] Martínez-Jiménez F, Movasati A, Brunner SR, Nguyen L, Priestley P, Cuppen E, et al. Pan-cancer whole-genome comparison of primary and metastatic solid tumours. Nature. 2023. 10.1038/s41586-023-06054-z. 37165194 10.1038/s41586-023-06054-z PMC10247378

[27] Dominiak A, Chełstowska B, Olejarz W, Nowicka G. Communication in the Cancer Microenvironment as a Target for Therapeutic Interventions. Cancers (Basel). 2020;12:1232. 32422889 10.3390/cancers12051232 PMC7281160

[28] Zheng G, Ma Y, Zou Y, Yin A, Li W, Dong D. HCMDB: the human cancer metastasis database. Nucleic Acids Res. 2018;46:D950–5. 29088455 10.1093/nar/gkx1008 PMC5753185

[29] Mohammadi S, Davila-Velderrain J, Kellis M. A multiresolution framework to characterize single-cell state landscapes. Nat Commun. 2020;11:5399. 33106496 10.1038/s41467-020-18416-6 PMC7588427

[30] Wei H, Li J, Xie M, Lei R, Hu B. Comprehensive analysis of metastasis-related genes reveals a gene signature predicting the survival of colon cancer patients. PeerJ. 2018;6: e5433. 30155352 10.7717/peerj.5433 PMC6108311

[31] Weinstein JN, Collisson EA, Mills GB, Shaw KRM, Ozenberger BA, Ellrott K, et al. The Cancer Genome Atlas Pan-Cancer analysis project. Nat Genet. 2013;45:1113–20. 24071849 10.1038/ng.2764 PMC3919969

[32] Andreatta M, Carmona SJ. UCell: Robust and scalable single-cell gene signature scoring. Comput Struct Biotechnol J. 2021;19:3796–8. 34285779 10.1016/j.csbj.2021.06.043 PMC8271111

[33] Adler O, Zait Y, Cohen N, Blazquez R, Doron H, Monteran L, et al. Reciprocal interactions between innate immune cells and astrocytes facilitate neuroinflammation and brain metastasis via lipocalin-2. Nat Cancer. 2023;4:401–18. 36797502 10.1038/s43018-023-00519-w

[34] Hu C, Yang K, Li M, Huang W, Zhang F, Wang H. Lipocalin 2: a potential therapeutic target for breast cancer metastasis. Onco Targets Ther. 2018;11:8099–106. 30519052 10.2147/OTT.S181223 PMC6239117

[35] Tian S, Chu Y, Hu J, Ding X, Liu Z, Fu D, et al. Tumour-associated neutrophils secrete AGR2 to promote colorectal cancer metastasis via its receptor CD98hc–xCT. Gut. 2022;71:2489–501. 35086885 10.1136/gutjnl-2021-325137

[36] Liu Y, Ge J, Chen Y, Liu T, Chen L, Liu C, et al. Combined Single-Cell and Spatial Transcriptomics Reveal the Metabolic Evolvement of Breast Cancer during Early Dissemination. Adv Sci. 2023;10:2205395. 10.1002/advs.202205395 PMC9951304 36594618

[37] Berglund E, Maaskola J, Schultz N, Friedrich S, Marklund M, Bergenstråhle J, et al. Spatial maps of prostate cancer transcriptomes reveal an unexplored landscape of heterogeneity. Nat Commun. 2018;9:2419. 29925878 10.1038/s41467-018-04724-5 PMC6010471

[38] Gulati GS, Sikandar SS, Wesche DJ, Manjunath A, Bharadwaj A, Berger MJ et al. Single-cell transcriptional diversity is a hallmark of developmental potential. Science (80- ) 2020; 367: 405–411. 10.1126/science.aax0249 PMC7694873 31974247

[39] Mei D, Zhu Y, Zhang L, Wei W. The Role of CTHRC1 in Regulation of Multiple Signaling and Tumor Progression and Metastasis. Mediators Inflamm. 2020;2020:1–13. 10.1155/2020/9578701 PMC7441421 32848510

[40] Zhang X-L, Hu L-P, Yang Q, Qin W-T, Wang X, Xu C-J, et al. CTHRC1 promotes liver metastasis by reshaping infiltrated macrophages through physical interactions with TGF-β receptors in colorectal cancer. Oncogene. 2021;40:3959–73. 33986509 10.1038/s41388-021-01827-0

[41] Li H, Liu W, Zhang X, Wang Y. Cancer-associated fibroblast-secreted collagen triple helix repeat containing-1 promotes breast cancer cell migration, invasiveness and epithelial-mesenchymal transition by activating the Wnt/β-catenin pathway. Oncol Lett. 2021;22:814. 34671428 10.3892/ol.2021.13075 PMC8503808

[42] Siegel MB, He X, Hoadley KA, Hoyle A, Pearce JB, Garrett AL, et al. Integrated RNA and DNA sequencing reveals early drivers of metastatic breast cancer. J Clin Invest. 2018;128:1371–83. 29480819 10.1172/JCI96153 PMC5873890

[43] Simeonov KP, Byrns CN, Clark ML, Norgard RJ, Martin B, Stanger BZ, et al. Single-cell lineage tracing of metastatic cancer reveals selection of hybrid EMT states. Cancer Cell. 2021;39:1150-1162.e9. 34115987 10.1016/j.ccell.2021.05.005 PMC8782207

[44] Xu K, Wang R, Xie H, Hu L, Wang C, Xu J, et al. Single-cell RNA sequencing reveals cell heterogeneity and transcriptome profile of breast cancer lymph node metastasis. Oncogenesis. 2021;10:66. 34611125 10.1038/s41389-021-00355-6 PMC8492772

[45] Cao EY, Ouyang JF, Rackham OJL. GeneSwitches: ordering gene expression and functional events in single-cell experiments. Bioinformatics. 2020;36:3273–5. 32058565 10.1093/bioinformatics/btaa099

[46] Huang Y, Rao A. Connections between TET proteins and aberrant DNA modification in cancer. Trends Genet. 2014;30:464–74. 25132561 10.1016/j.tig.2014.07.005 PMC4337960

[47] Pham D, Tan X, Xu J, Grice LF, Lam PY, Raghubar A et al. stLearn: integrating spatial location, tissue morphology and gene expression to find cell types, cell-cell interactions and spatial trajectories within undissociated tissues. bioRxiv 2020; : 2020.05.31.125658.

[48] Li G, Jiang W, Kang Y, Yu X, Zhang C, Feng Y. High expression of collagen 1A2 promotes the proliferation and metastasis of esophageal cancer cells. Ann Transl Med. 2020;8:1672–1672. 33490184 10.21037/atm-20-7867 PMC7812173

[49] Bergfeld SA, DeClerck YA. Bone marrow-derived mesenchymal stem cells and the tumor microenvironment. Cancer Metastasis Rev. 2010;29:249–61. 20411303 10.1007/s10555-010-9222-7

[50] Bussard KM, Mutkus L, Stumpf K, Gomez-Manzano C, Marini FC. Tumor-associated stromal cells as key contributors to the tumor microenvironment. Breast Cancer Res. 2016;18:84. 27515302 10.1186/s13058-016-0740-2 PMC4982339

[51] Jin S, Guerrero-Juarez CF, Zhang L, Chang I, Ramos R, Kuan C-H, et al. Inference and analysis of cell-cell communication using Cell Chat. Nat Commun. 2021;12:1088. 33597522 10.1038/s41467-021-21246-9 PMC7889871

[52] Zhang Y, Wang X. Targeting the Wnt/β-catenin signaling pathway in cancer. J Hematol Oncol. 2020;13:165. 33276800 10.1186/s13045-020-00990-3 PMC7716495

[53] Hoang BH, Kubo T, Healey JH, Sowers R, Mazza B, Yang R, et al. Expression of LDL receptor-related protein 5 (LRP5) as a novel marker for disease progression in high-grade osteosarcoma. Int J Cancer. 2004;109:106–11. 14735475 10.1002/ijc.11677

[54] Dimitrov D, Türei D, Garrido-Rodriguez M, Burmedi PL, Nagai JS, Boys C, et al. Comparison of methods and resources for cell-cell communication inference from single-cell RNA-Seq data. Nat Commun. 2022;13:3224. 35680885 10.1038/s41467-022-30755-0 PMC9184522

[55] Wang Z, Zhao T, Zhang S, Wang J, Chen Y, Zhao H, et al. The Wnt signaling pathway in tumorigenesis, pharmacological targets, and drug development for cancer therapy. Biomark Res. 2021;9:68. 34488905 10.1186/s40364-021-00323-7 PMC8422786

[56] Ashburn TT, Thor KB. Drug repositioning: identifying and developing new uses for existing drugs. Nat Rev Drug Discov. 2004;3:673–83. 15286734 10.1038/nrd1468

[57] Pushpakom S, Iorio F, Eyers PA, Escott KJ, Hopper S, Wells A, et al. Drug repurposing: progress, challenges and recommendations. Nat Rev Drug Discov. 2019;18:41–58. 30310233 10.1038/nrd.2018.168

[58] He B, Xiao Y, Liang H, Huang Q, Du Y, Li Y, et al. ASGARD is A Single-cell Guided Pipeline to Aid Repurposing of Drugs. Nat Commun. 2023;14:993. 36813801 10.1038/s41467-023-36637-3 PMC9945835

[59] Ritchie ME, Phipson B, Wu D, Hu Y, Law CW, Shi W, et al. limma powers differential expression analyses for RNA-sequencing and microarray studies. Nucleic Acids Res. 2015;43:e47–e47. 25605792 10.1093/nar/gkv007 PMC4402510

[60] Pan C-H, Chang Y-F, Lee M-S, Wen B-C, Ko J-C, Liang S-K, et al. Vorinostat enhances the cisplatin-mediated anticancer effects in small cell lung cancer cells. BMC Cancer. 2016;16:857. 27821078 10.1186/s12885-016-2888-7 PMC5100277

[61] Wawruszak A, Borkiewicz L, Okon E, Kukula-Koch W, Afshan S, Halasa M. Vorinostat (SAHA) and Breast Cancer: An Overview. Cancers (Basel). 2021;13:4700. 34572928 10.3390/cancers13184700 PMC8468501

[62] Bubna A. Vorinostat-An overview. Indian J Dermatol. 2015;60:419. 26288427 10.4103/0019-5154.160511 PMC4533557

[63] Linden HM, Kurland BF, Link J, Novakova A, Chai X, Specht JM, et al. A phase II clinical trial of HDACi (vorinostat) and AI therapy in breast cancer with molecular imaging correlates. J Clin Oncol. 2014;32:556.

[64] Wang Y, Janku F, Piha-Paul S, Hess K, Broaddus R, Liu L, et al. Phase I studies of vorinostat with ixazomib or pazopanib imply a role of antiangiogenesis-based therapy for TP53 mutant malignancies. Sci Rep. 2020;10:3080. 32080210 10.1038/s41598-020-58366-z PMC7033174

[65] Kim MJ, Huang Y, Park J-I. Targeting Wnt Signaling for Gastrointestinal Cancer Therapy: Present and Evolving Views. Cancers (Basel). 2020;12:3638. 33291655 10.3390/cancers12123638 PMC7761926

[66] Kamimoto K, Stringa B, Hoffmann CM, Jindal K, Solnica-Krezel L, Morris SA. Dissecting cell identity via network inference and in silico gene perturbation. Nature. 2023;614:742–51. 36755098 10.1038/s41586-022-05688-9 PMC9946838

[67] Ma J-B, Bai J-Y, Zhang H-B, Jia J, Shi Q, Yang C, et al. KLF5 inhibits STAT3 activity and tumor metastasis in prostate cancer by suppressing IGF1 transcription cooperatively with HDAC1. Cell Death Dis. 2020;11:466. 32546700 10.1038/s41419-020-2671-1 PMC7297795

[68] Meissl K, Macho-Maschler S, Müller M, Strobl B. The good and the bad faces of STAT1 in solid tumours. Cytokine. 2017;89:12–20. 26631912 10.1016/j.cyto.2015.11.011

[69] Gao Y, Gan K, Liu K, Xu B, Chen M. SP1 Expression and the Clinicopathological Features of Tumors: A Meta-Analysis and Bioinformatics Analysis. Pathol Oncol Res 2021; 27. 10.3389/pore.2021.581998. 10.3389/pore.2021.581998 PMC8262197 34257529

[70] Samaržija I. Wnt Signaling Pathway Is among the Drivers of Liver Metastasis. Livers. 2021;1:180–200.

[71] Su J, Wu S, Wu H, Li L, Guo T. CD44 is functionally crucial for driving lung cancer stem cells metastasis through Wnt/β-catenin-FoxM1-Twist signaling. Mol Carcinog. 2016;55:1962–73. 26621583 10.1002/mc.22443

[72] Ueno K, Hirata H, Hinoda Y, Dahiya R. Frizzled homolog proteins, microRNAs and Wnt signaling in cancer. Int J Cancer. 2013;132:1731–40. 22833265 10.1002/ijc.27746 PMC3940357

[73] Shen C, Nayak A, Melendez RA, Wynn DT, Jackson J, Lee E, et al. Casein Kinase 1α as a Regulator of Wnt-Driven Cancer. Int J Mol Sci. 2020;21:5940. 32824859 10.3390/ijms21165940 PMC7460588

[74] Lu Y, Qin H, Jiang B, Lu W, Hao J, Cao W, et al. KLF2 inhibits cancer cell migration and invasion by regulating ferroptosis through GPX4 in clear cell renal cell carcinoma. Cancer Lett. 2021;522:1–13. 34520818 10.1016/j.canlet.2021.09.014

[75] Wang B, Liu M, Song Y, Li C, Zhang S, Ma L. KLF2 Inhibits the Migration and Invasion of Prostate Cancer Cells by Downregulating MMP2. Am J Mens Health. 2019;13:155798831881690. 10.1177/1557988318816907 PMC6775556 30520325

[76] Malek A, Núñez L-E, Magistri M, Brambilla L, Jovic S, Carbone GM, et al. Modulation of the Activity of Sp Transcription Factors by Mithramycin Analogues as a New Strategy for Treatment of Metastatic Prostate Cancer. PLoS ONE. 2012;7: e35130. 22545098 10.1371/journal.pone.0035130 PMC3334962

[77] Crosas-Molist E, Samain R, Kohlhammer L, Orgaz JL, George SL, Maiques O, et al. Rho GTPase signaling in cancer progression and dissemination. Physiol Rev. 2022;102:455–510. 34541899 10.1152/physrev.00045.2020

[78] Humphries B, Wang Z, Yang C. Rho GTPases: Big Players in Breast Cancer Initiation. Metastasis and Therapeutic Responses Cells. 2020;9:2167. 32992837 10.3390/cells9102167 PMC7600866

[79] Crosas-Molist E, Bertran E, Rodriguez-Hernandez I, Herraiz C, Cantelli G, Fabra À, et al. The NADPH oxidase NOX4 represses epithelial to amoeboid transition and efficient tumour dissemination. Oncogene. 2017;36:3002–14. 27941881 10.1038/onc.2016.454 PMC5354266

[80] Sanz-Moreno V, Gadea G, Ahn J, Paterson H, Marra P, Pinner S, et al. Rac Activation and Inactivation Control Plasticity of Tumor Cell Movement. Cell. 2008;135:510–23. 18984162 10.1016/j.cell.2008.09.043

[81] Calvo F, Sanz-Moreno V, Agudo-Ibáñez L, Wallberg F, Sahai E, Marshall CJ, et al. RasGRF suppresses Cdc42-mediated tumour cell movement, cytoskeletal dynamics and transformation. Nat Cell Biol. 2011;13:819–26. 21685891 10.1038/ncb2271

[82] Cantelli G, Orgaz JL, Rodriguez-Hernandez I, Karagiannis P, Maiques O, Matias-Guiu X, et al. TGF-β-Induced Transcription Sustains Amoeboid Melanoma Migration and Dissemination. Curr Biol. 2015;25:2899–914. 26526369 10.1016/j.cub.2015.09.054 PMC4651903

[83] Liu S, Shen M, Hsu E-C, Zhang CA, Garcia-Marques F, Nolley R, et al. Discovery of PTN as a serum-based biomarker of pro-metastatic prostate cancer. Br J Cancer. 2021;124:896–900. 33288843 10.1038/s41416-020-01200-0 PMC7921397

[84] Jiang NY, Woda BA, Banner BF, Whalen GF, Dresser KA, Lu D. Sp1, a New Biomarker That Identifies a Subset of Aggressive Pancreatic Ductal Adenocarcinoma. Cancer Epidemiol Biomarkers Prev. 2008;17:1648–52. 18628415 10.1158/1055-9965.EPI-07-2791

[85] Yao JC, Wang L, Wei D, Gong W, Hassan M, Wu T-T, et al. Association between Expression of Transcription Factor Sp1 and Increased Vascular Endothelial Growth Factor Expression, Advanced Stage, and Poor Survival in Patients with Resected Gastric Cancer. Clin Cancer Res. 2004;10:4109–17. 15217947 10.1158/1078-0432.CCR-03-0628

[86] Hsu M-C, Chang H-C, Hung W-C. HER-2/neu Represses the Metastasis Suppressor RECK via ERK and Sp Transcription Factors to Promote Cell Invasion. J Biol Chem. 2006;281:4718–25. 16377629 10.1074/jbc.M510937200

[87] Ma Z, Chang MJ, Shah R, Adamski J, Zhao X, Benveniste EN. Brg-1 Is Required for Maximal Transcription of the Human Matrix Metalloproteinase-2 Gene. J Biol Chem. 2004;279:46326–34. 15317818 10.1074/jbc.M405438200

[88] Nam E-H, Lee Y, Park Y-K, Lee JW, Kim S. ZEB2 upregulates integrin α5 expression through cooperation with Sp1 to induce invasion during epithelial–mesenchymal transition of human cancer cells. Carcinogenesis. 2012;33:563–71. 22227038 10.1093/carcin/bgs005

[89] Monteleone E, Orecchia V, Corrieri P, Schiavone D, Avalle L, Moiso E, et al. SP1 and STAT3 Functionally Synergize to Induce the RhoU Small GTPase and a Subclass of Non-canonical WNT Responsive Genes Correlating with Poor Prognosis in Breast Cancer. Cancers (Basel). 2019;11:101. 30654518 10.3390/cancers11010101 PMC6356433

[90] Mir R, Sharma A, Pradhan SJ, Galande S. Regulation of Transcription Factor SP1 by the β -Catenin Destruction Complex Modulates Wnt Response. Mol Cell Biol 2018; 38. 10.1128/MCB.00188-18. 10.1128/MCB.00188-18 PMC6206460 30181396

[91] Haga RB, Ridley AJ. Rho GTPases: Regulation and roles in cancer cell biology. Small GTPases. 2016;7:207–21. 27628050 10.1080/21541248.2016.1232583 PMC5129894

[92] Rodriguez-Hernandez I, Cantelli G, Bruce F, Sanz-Moreno V. Rho, ROCK and actomyosin contractility in metastasis as drug targets. F1000Research 2016; 5: 783. 10.12688/f1000research.7909.1 PMC4856114 27158478

[93] Clayton NS, Ridley AJ. Targeting Rho GTPase Signaling Networks in Cancer. Front Cell Dev Biol 2020; 8. 10.3389/fcell.2020.00222. 10.3389/fcell.2020.00222 PMC7145979 32309283

[94] Chen C, Zhou Y, Zhou Z, Sun X, Otto KB, Uht RM, et al. Regulation of KLF5 involves the Sp1 transcription factor in human epithelial cells. Gene. 2004;330:133–42. 15087132 10.1016/j.gene.2004.01.014

[95] Mann BS, Johnson JR, Cohen MH, Justice R, Pazdur R. FDA approval summary: vorinostat for treatment of advanced primary cutaneous T-cell lymphoma. Oncologist. 2007;12:1247–52. 17962618 10.1634/theoncologist.12-10-1247

[96] Siegel D, Hussein M, Belani C, Robert F, Galanis E, Richon VM, et al. Vorinostat in solid and hematologic malignancies. J Hematol Oncol. 2009;2:31. 19635146 10.1186/1756-8722-2-31 PMC2731787

[97] Palmieri D, Lockman PR, Thomas FC, Hua E, Herring J, Hargrave E, et al. Vorinostat inhibits brain metastatic colonization in a model of triple-negative breast cancer and induces DNA double-strand breaks. Clin Cancer Res. 2009;15:6148–57. 19789319 10.1158/1078-0432.CCR-09-1039 PMC7356672

[98] Wawruszak A, Luszczki J, Halasa M, Okon E, Landor S, Sahlgren C et al. Sensitization of MCF7 Cells with High Notch1 Activity by Cisplatin and Histone Deacetylase Inhibitors Applied Together. Int J Mol Sci 2021; 22. 10.3390/ijms22105184. 10.3390/ijms22105184 PMC8153599 34068438

[99] Nouri Emamzadeh F, Word B, Cotton E, Hawkins A, Littlejohn K, Moore R, et al. Modulation of Estrogen α and Progesterone Receptors in Triple Negative Breast Cancer Cell Lines: The Effects of Vorinostat and Indole-3-Carbinol In Vitro. Anticancer Res. 2020;40:3669–83. 32620606 10.21873/anticanres.14356

[100] Srivastava RK, Kurzrock R, Shankar S. MS-275 Sensitizes TRAIL-Resistant Breast Cancer Cells, Inhibits Angiogenesis and Metastasis, and Reverses Epithelial-Mesenchymal Transition In vivo. Mol Cancer Ther. 2010;9:3254–66. 21041383 10.1158/1535-7163.MCT-10-0582

[101] Cheng H-W, Liang Y-H, Kuo Y-L, Chuu C-P, Lin C-Y, Lee M-H, et al. Identification of thioridazine, an antipsychotic drug, as an antiglioblastoma and anticancer stem cell agent using public gene expression data. Cell Death Dis. 2015;6:e1753–e1753. 25950483 10.1038/cddis.2015.77 PMC4669717

[102] Chu C-W, Ko H-J, Chou C-H, Cheng T-S, Cheng H-W, Liang Y-H et al. Thioridazine Enhances P62-Mediated Autophagy and Apoptosis Through Wnt/β-Catenin Signaling Pathway in Glioma Cells. Int J Mol Sci 2019; 20. 10.3390/ijms20030473. 10.3390/ijms20030473 PMC6386927 30678307

[103] Wang Y, Xia L, Lin J, Gong L, Xia Y, Xu Y, et al. Thioridazine combined with carboplatin results in synergistic inhibition of triple negative breast cancer by targeting cancer stem cells. Transl Oncol. 2022;26: 101549. 36191461 10.1016/j.tranon.2022.101549 PMC9530598

[104] Song Y, Li L, Chen J, Chen H, Cui B, Feng Y, et al. Thioridazine hydrochloride: an antipsychotic agent that inhibits tumor growth and lung metastasis in triple-negative breast cancer via inducing G0/G1 arrest and apoptosis. Cell Cycle. 2020;19:3521–33. 33315498 10.1080/15384101.2020.1850969 PMC7781661

[105] Yin L, Gao Y, Zhang X, Wang J, Ding D, Zhang Y, et al. Niclosamide sensitizes triple-negative breast cancer cells to ionizing radiation in association with the inhibition of Wnt/β-catenin signaling. Oncotarget. 2016;7:42126–38. 27363012 10.18632/oncotarget.9704 PMC5173121

[106] Guo Y, Zhu H, Xiao Y, Guo H, Lin M, Yuan Z, et al. Correction: The anthelmintic drug niclosamide induces GSK-β-mediated β-catenin degradation to potentiate gemcitabine activity, reduce immune evasion ability and suppress pancreatic cancer progression. Cell Death Dis. 2022;13:366. 35440080 10.1038/s41419-022-04705-z PMC9018699

[107] Lu D, Choi MY, Yu J, Castro JE, Kipps TJ, Carson DA. Salinomycin inhibits Wnt signaling and selectively induces apoptosis in chronic lymphocytic leukemia cells. Proc Natl Acad Sci U S A. 2011;108:13253–7. 21788521 10.1073/pnas.1110431108 PMC3156152

[108] Tang Q-L, Zhao Z-Q, Li J-C, Liang Y, Yin J-Q, Zou C-Y, et al. Salinomycin inhibits osteosarcoma by targeting its tumor stem cells. Cancer Lett. 2011;311:113–21. 21835542 10.1016/j.canlet.2011.07.016

[109] Canesin G, Evans-Axelsson S, Hellsten R, Krzyzanowska A, Prasad CP, Bjartell A, et al. Treatment with the WNT5A-mimicking peptide Foxy-5 effectively reduces the metastatic spread of WNT5A-low prostate cancer cells in an orthotopic mouse model. PLoS ONE. 2017;12: e0184418. 28886116 10.1371/journal.pone.0184418 PMC5590932

