# Supplementary material for: Pan-cancer drivers of metastasis
Source: Mol Cancer. 2025 Jan 2;24:2. doi: 10.1186/s12943-024-02182-w (PMC11697158; doi:10.1186/s12943-024-02182-w)

Supplementary Figure 1

Lusby et al

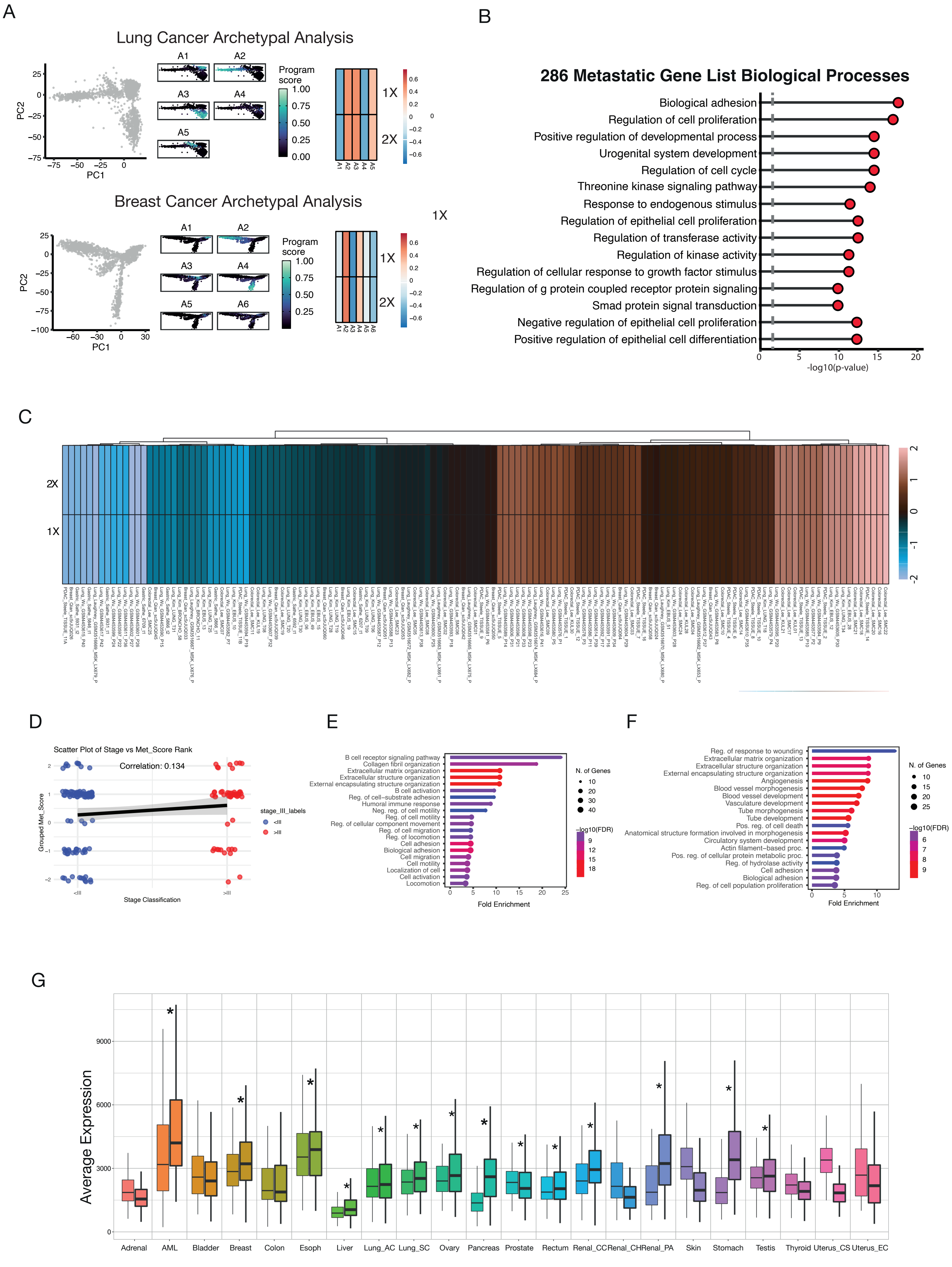

Supplementary Figure 2 Lusby et al

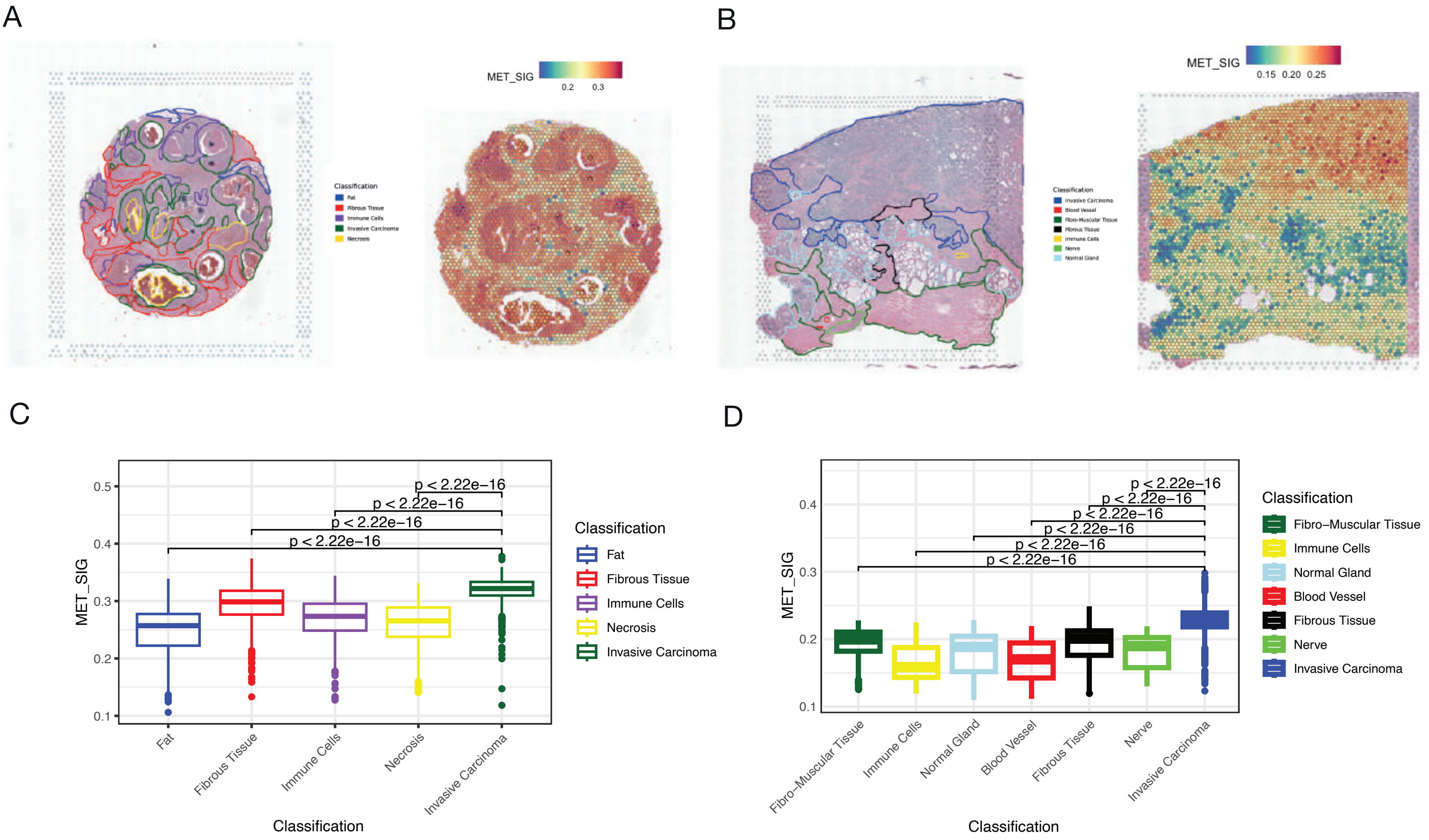

Supplementary Figure 3

Lusby et al

A

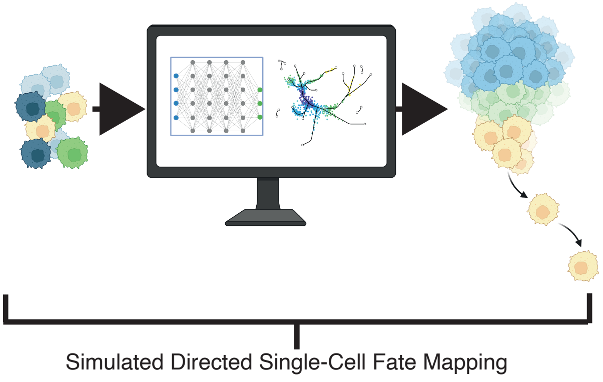

B

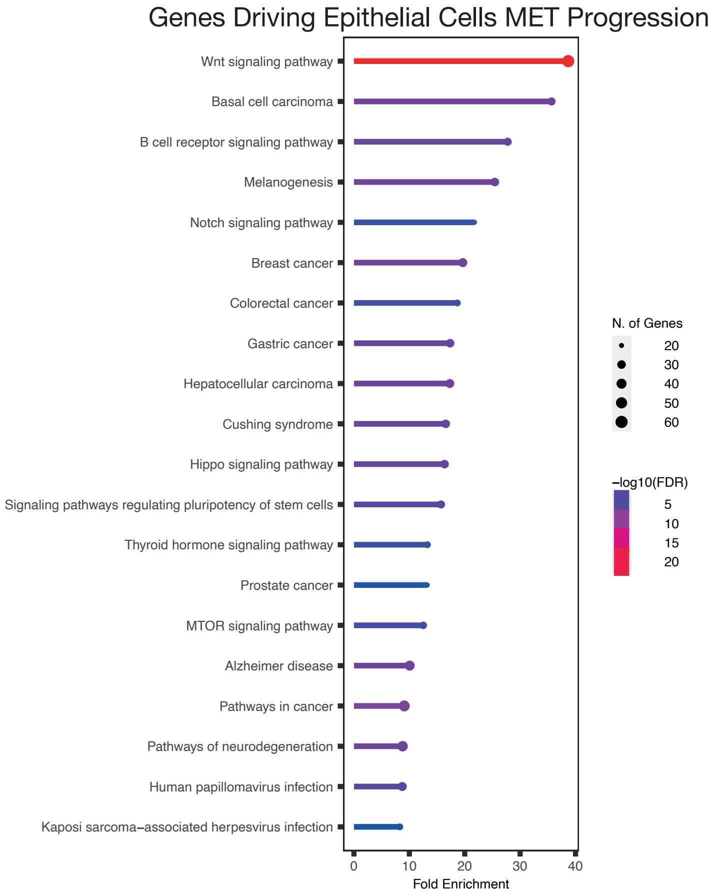

C

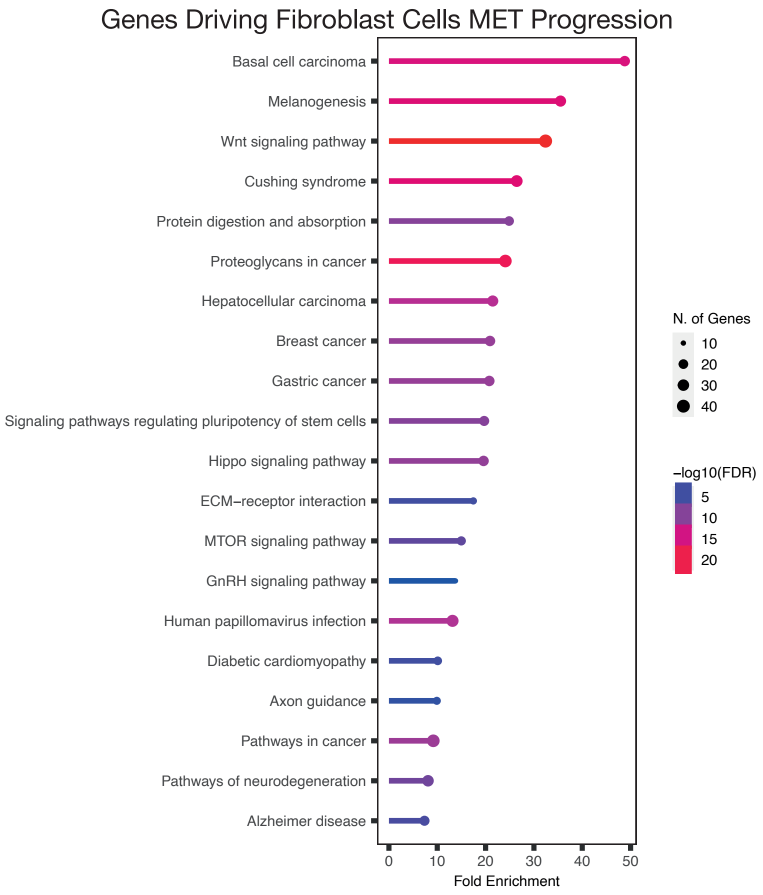

Supplementary Figure 4

Lusby et al

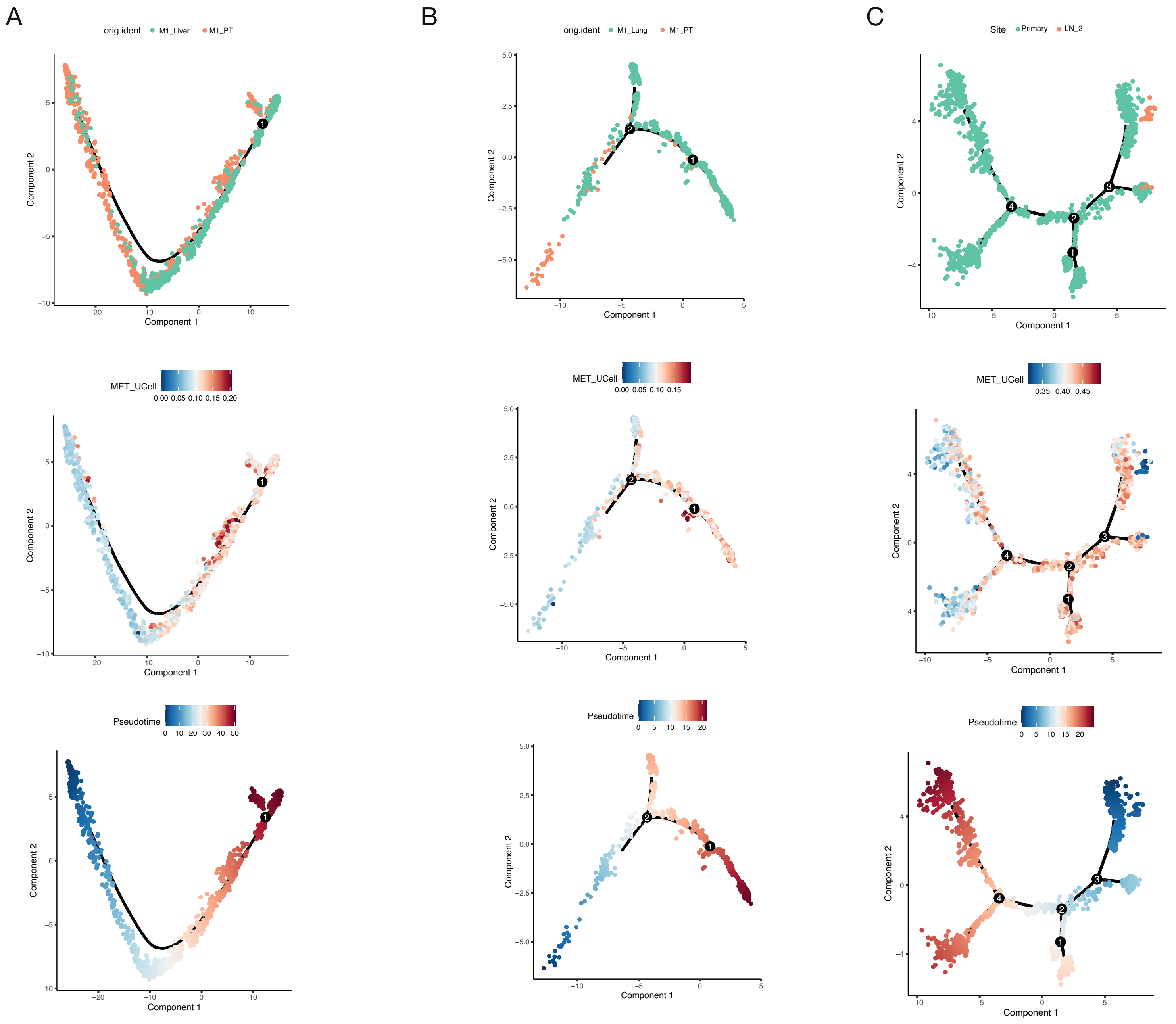

Supplementary Figure 5 Lusby et al

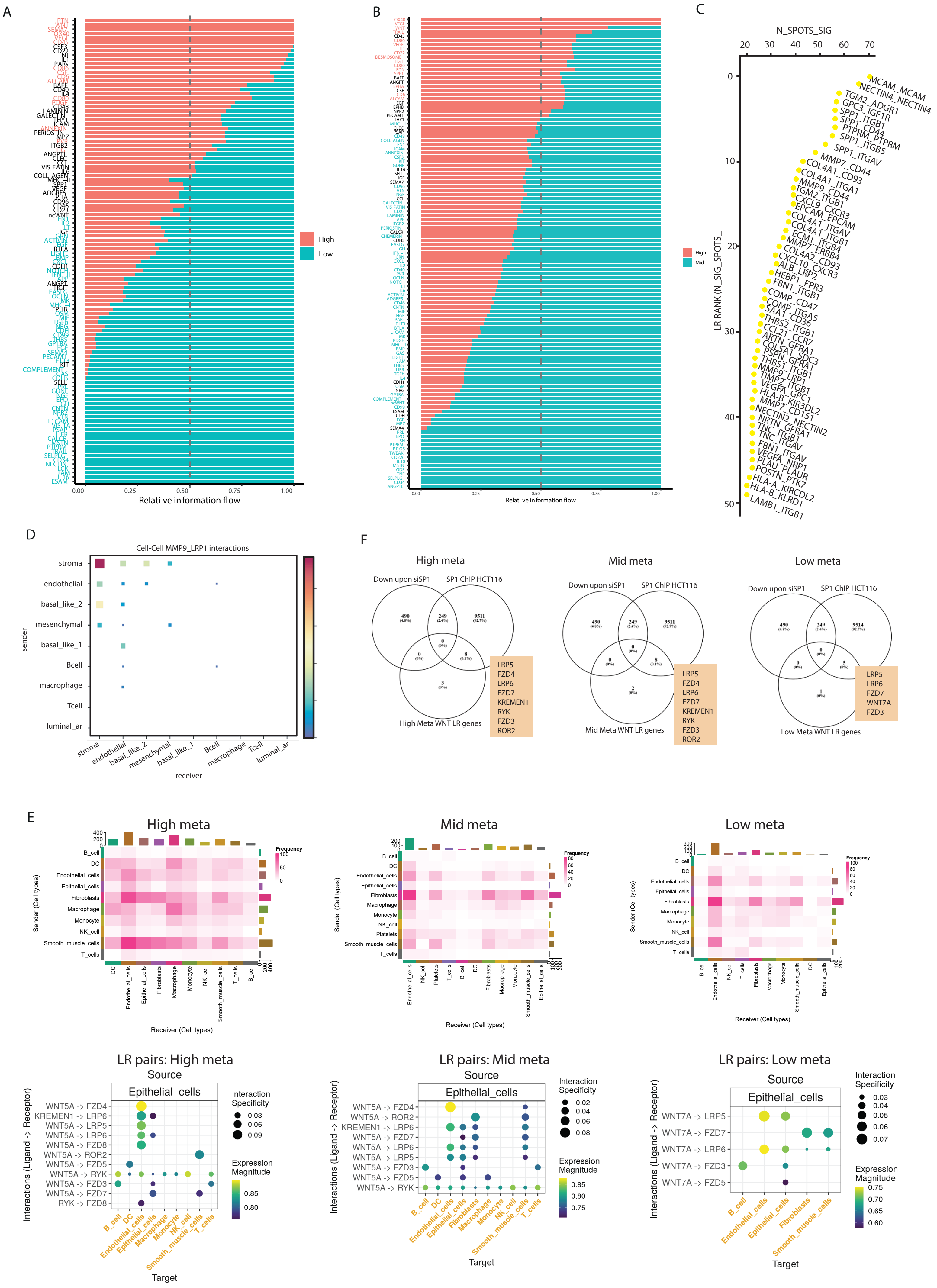

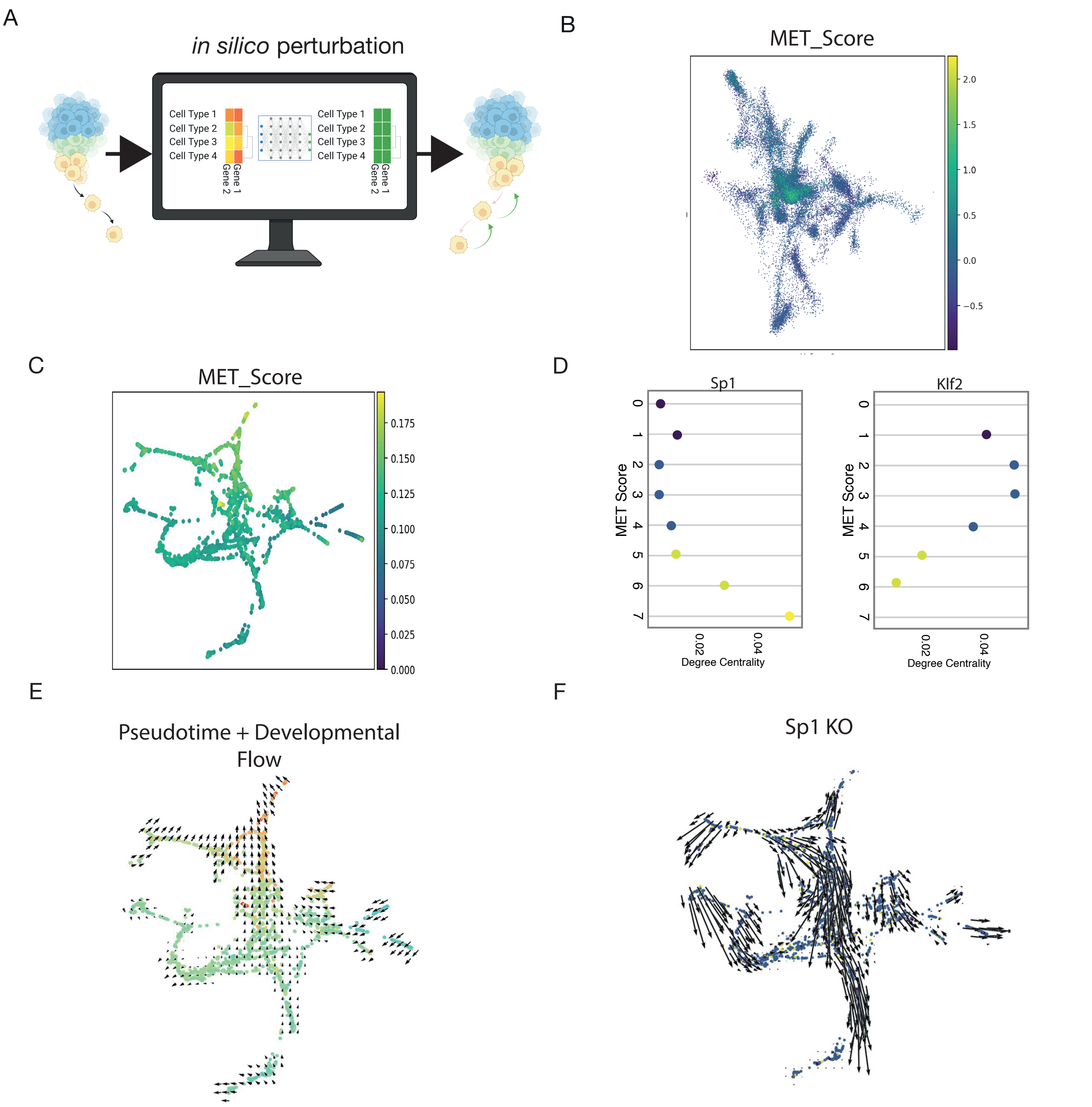
A
in silico perturbation
Cell Type 1
Cell Type 2
Cell Type 3
Cell Type 4
Gene 1
Gene 2
B
MET_Score
2.0
1.5
1.0
0.5
0.0
−0.5
C
MET_Score
0.175
0.150
0.125
0.100
0.075
0.050
0.025
0.000
D
Sp1
Klf2
MET Score
0
1
2
3
4
5
6
7
0.02
0.04
Degree Centrality
E
Pseudotime + Developmental Flow
F
Sp1 KO

Supplementary Figure 7 Lusby et al

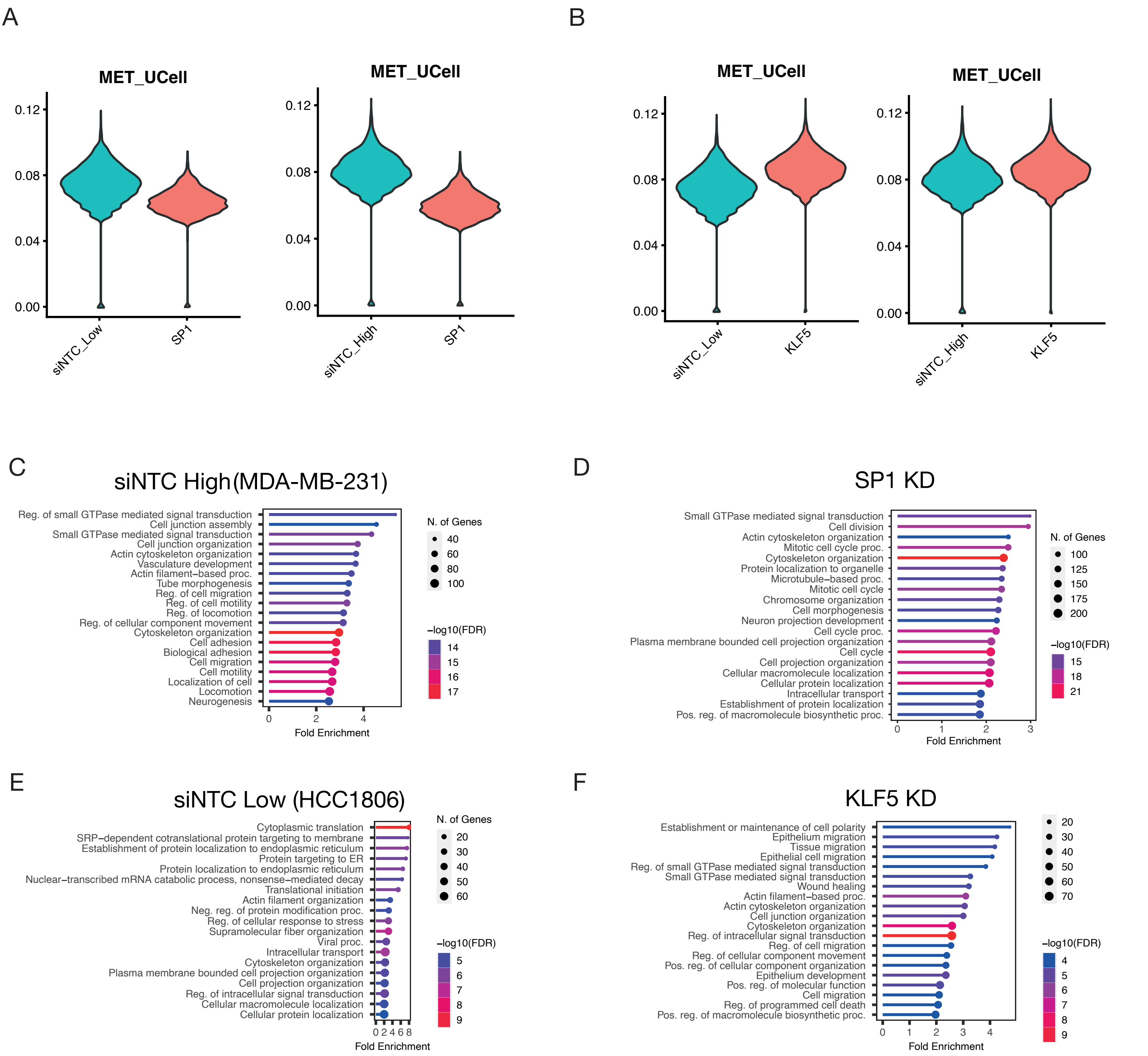

Supplementary Figure S8

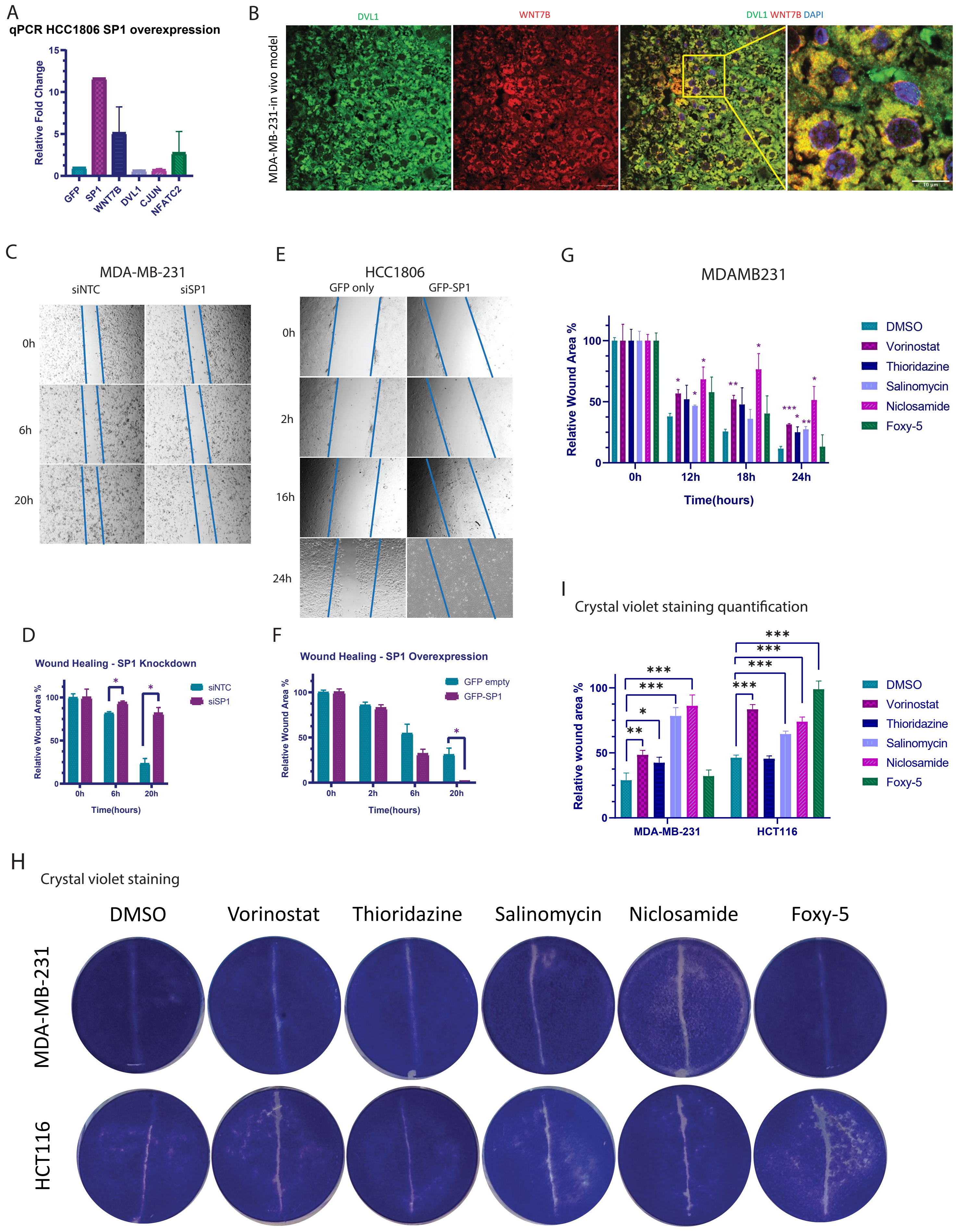

Supplement: Supplementary file 1 — Supplementary Material 1. Supplementary Figure 1. scRNA-seq of each cancer type and scoring of each patient (A) Archetypal analysis of lung and breast cancer patients, with each archetype scored using both gene lists. (B) Gene Ontology (GO) enrichment results for the 286 metastatic gene signature. (C) Stratification of cancer patients based on metastatic scoring. (D) Correlation between the 286-gene signature scores and patient stages (r = 0.134). (E) GO enrichment analysis of the 177-gene signature. (F) GO enrichment analysis of the 109-gene set. (G) Average expression levels of the refined gene signature across TCGA pan-cancer datasets, where * indicates significantly higher expression in tumours compared to normal tissues. Supplementary Figure 2. Spatial transcriptomics scoring and pseudotime analysis (A) Spatial transcriptomic map of a breast cancer patient scored for metastatic potential using a 177-gene signature with UCell. (B) Spatial transcriptomic map of a prostate cancer patient scored for metastatic potential using the same 177-gene signature with UCell. (C) Expression patterns of the metastatic signature across invasive carcinoma and other annotated regions in breast cancer spatial transcriptomic data. (D) Expression patterns of the metastatic signature across invasive carcinoma and other annotated regions in prostate cancer spatial transcriptomic data. Supplementary Figure 3. Genes driving cell type specific metastatic progression (A) Schematic overview of the CellRank method, illustrating how cells are arranged and mapped based on their cellular fate trajectories toward a common endpoint. (B) KEGG pathway enrichment analysis of the top genes driving metastatic progression in epithelial cells. (C) KEGG pathway enrichment analysis of the top genes driving metastatic progression in fibroblast cells. Supplementary Figure 4. Extended pseudotime analysis (A-C) pseudotime analysis with metastatic scoring of cells using UCell. Supplementary Figure 5. Cell-Cel [file 12943_2024_2182_MOESM1_ESM.pdf]
